# Targeting NF-κB Signaling with Natural Products: A Promising Therapeutic Strategy for Cardiovascular Diseases

**DOI:** 10.3390/biom16040491

**Published:** 2026-03-25

**Authors:** Rui Liu, Wencong Liu, Ling Dong, Shuang Ma, Baojun Xu

**Affiliations:** 1 College of Food and Pharmaceutical Engineering, Wuzhou University, Wuzhou 543002, China; rayfayliuruifei@163.com (R.L.);; 2 Guangxi Liupao Tea Modern Industry College, Wuzhou University, Wuzhou 543002, China; 3 Food Science and Technology Program, Department of Life Sciences, Beijing Normal-Hong Kong Baptist University, Zhuhai 519087, China

**Keywords:** cardiovascular diseases, natural products, NF-κB signaling pathway, inflammation, oxidative stress, vascular remodeling, molecular mechanisms

## Abstract

Cardiovascular diseases (CVDs) remain the primary cause of human morbidity and mortality in the world. Inflammation, oxidative stress, and vascular remodeling are the key factors that make CVDs worse. The nuclear factor κB (NF-κB) signaling pathway is a major regulator in the progression of CVDs. NF-κB activates wrongly, induces the secretion of pro-inflammatory cytokines (including TNF-α, IL-6, and IL-1β), and enhances reactive oxygen species (ROS) generation. These accelerate endothelial dysfunction, myocardial damage, and atherosclerotic plaque development. Natural products are structurally diverse, multi-targeted, and low toxicity. They offer a promising way to prevent and treat cardiovascular disease by modulating the NF-κB signaling pathway. This review summarizes the recent studies about using natural products (including flavonoids, terpenoids, alkaloids, polyphenols, and polysaccharides) to treat CVDs through the NF-κB pathway, with a critical analysis of evidence strength according to CVDs indication (atherosclerosis, myocardial ischemia/reperfusion injury, pulmonary arterial hypertension, etc.) and study type (in vitro, in vivo animal, and human clinical research). We detail their molecular mechanisms, such as inhibiting the nuclear translocation of NF-κB p65, downregulating IκB phosphorylation, blocking upstream signaling (e.g., TLR4/MyD88, PI3K/Akt, MAPK), and affecting with other pathways (e.g., Nrf2/HO-1, SIRT1) to reduce inflammation and oxidative stress together. We also detail the effects of these natural products in various CVDs models, including atherosclerosis, hypertension, myocardial ischemia/reperfusion injury, diabetic cardiomyopathy, and pulmonary arterial hypertension, highlighting the characteristics of their treatments. Finally, we discuss the challenges of bringing natural products into the clinic and share some ideas to solve difficulties, with an in-depth critical analysis of the translational bottlenecks (poor bioavailability, unclear structure–activity relationships, incomplete mechanistic elucidation, and lack of large-scale clinical trials) and their underlying causes across different natural product classes. In summary, this review offers new perspectives on developing natural product-based therapies targeting the NF-κB signaling pathway for CVDs. It offers useful references for both preclinical studies and clinical applications.

## 1. Introduction

Cardiovascular diseases (CVDs) include atherosclerosis, myocardial infarction, hypertension, pulmonary arterial hypertension (PAH), diabetic cardiomyopathy (DCM), and septic cardiomyopathy. It remains the major cause of mortality and disease burden in the world. From 1990 to 2023, the number of CVD deaths increased to 19.2 million, while the number of CVD patients increased to 626 million [1]. Despite significant major clinical progress, including statins, antiplatelet drugs, and revascularization therapies, there are lots of questions, such as adverse effects, drug resistance, and single target issues. These questions need to be solved. Increasingly, evidence indicates that chronic inflammation and oxidative stress are the major factors of CVDs, and the nuclear factor-κB (NF-κB) signaling pathway is a primary regulator [2,3,4]. The NF-κB signaling pathway has been studied for over forty years. The scientists found it affects inflammation, immune regulation, and tumor micro-environments and tried different methods from IKK inhibitors to CAR-T cell therapy [5].

The NF-κB family has five main proteins: p65 (RelA), p50, p52, RelB, and c-Rel. These proteins work together to turn genes on. They mostly affect genes for pro-inflammatory cytokines, adhesion molecules, and proteins that control cell survival and death. NF-κB has two pathways. One is the classic canonical pathway, centered on IKK, IκB, and the p65/p50 complex. The other is the non-canonical pathway [5,6]. In CVDs, NF-κB can be activated by a lot of factors, such as oxidized low-density lipoprotein (ox-LDL), angiotensin II (Ang II), lipopolysaccharide (LPS), ischemia/reperfusion (I/R) injury, and high glucose [7,8,9]. For example, in atherosclerosis, NF-κB induces macrophage foam cell formation and endothelial inflammation [10], while, in hypertension, it induces inflammation in the hypothalamic paraventricular nucleus [7]. In myocardial I/R injury, excessive NF-κB activation induces inflammatory infiltration and cardiomyocyte apoptosis [11]. In PAH, activated NF-κB releases inflammatory cytokines, regulates the expression of remodeling-related molecules, and mediates pulmonary vascular and right ventricular remodeling [12], and, in diabetic cardiomyopathy, it causes myocardial fibrosis and dysfunction through regulating inflammatory expression [13]. So, targeting and inhibiting the NF-κB pathway has become a rational way for treating cardiovascular diseases. NF-κB is a key target for treating inflammatory cardiovascular conditions. Pimobendan and sodium-glucose cotransporter 2 inhibitors can promote anti-inflammation by inhibiting NF-κB in clinic, which may have beneficial effects in CVDs [14].

Many natural products, such as resveratrol and costunolide, can exhibit anti-inflammatory effects by targeting the NF-κB signaling pathway. They regulate inflammatory and antioxidant pathways or inhibit IKKβ and block NF-κB activation [15,16]. Costunolide has sufficient evidence from multiple ApoE-/- mouse models confirming its anti-atherosclerotic efficacy via direct IKKβ binding [16].

These natural products have been shown to affect NF-κB signaling in different ways, such as affecting IκBα conformational change [16]; inhibiting p65 nuclear translocation [8]; binding and inhibiting Src, AKT1, and COX-2 directly [17]; reducing inflammation and oxidative stress [18]; inhibiting TLR4, p65 TNF-α, and IL-1β; binding HMGB1; and, finally, inducing inflammatory responses [19,20]. For example, uteolin shows sufficient evidence for hypertension in SHR models by inhibiting NF-κB-mediated hypothalamic inflammation [7], with moderate support for preeclampsia. Quercetin has moderate evidence in various CVD models via NF-κB suppression [21], while astragaloside IV displays sufficient evidence against sepsis-induced cardiac dysfunction [22]. Colchicine presents moderate evidence for atherosclerosis and myocardial infarction [23]. Among these, atherosclerosis and myocardial I/R injury are the most well-validated indications. These findings support the therapeutic potential of natural products as NF-κB inhibitors for CVDs, with atherosclerosis and myocardial I/R injury being the most well-studied indications with the highest cumulative evidence across natural product classes.

Although preclinical evidence has increased, the challenges of introducing natural products into clinical practice still exist, including poor bioavailability, unclear structure–activity relationships, and incomplete mechanistic elucidation. This review stratifies each compound by CVD indication, characterizes evidence strength based on study type, and compares the translational potential across different natural product classes. As an example, flavonoids such as kaempferol and baicalin exhibit strong NF-κB inhibitory effects in cardiovascular abnormalities or atherosclerosis [24,25]. But their low water solubility and fast metabolism often limit their efficacy [26]. Meanwhile, the interactions between NF-κB and other key signaling pathways in CVDs, including Nrf2/HO-1, PI3K/Akt, MAPK, or SIRT1, should be further studied [11,27,28]. Moreover, there is a key problem in the lack of large-scale clinical trials to verify the effects of natural products in CVDs [2,3,15], with only a few compounds (e.g., resveratrol, sodium tanshinone IIA sulfonate) having even small-scale human clinical data, and most remaining in preclinical stages with varying levels of evidential robustness.

This review summarizes the effects of natural products (flavonoids, terpenoids, alkaloids, polysaccharides, lignans, saponins) on CVDs through the NF-κB pathway. We pay attention to their molecular mechanisms, pharmacological data from both in vitro and in vivo studies, and structure–activity relationships. We review the progress of the delivery systems, including nanocarriers, targeted modification strategies, and biomimetic nanoparticles. We also review the progress of clinical translation. We summarize the current challenges and provide ideas for future research. This review aims to provide a theoretical basis for the application of natural products in the treatment of CVDs through the NF-κB pathway.

## 2. The Roles of NF-κB Pathway in CVDs

The NF-κB family consists of several transcription factors, including RelA (also known as p65), RelB, c-Rel, p50 (derived from NF-κB1/p105), and p52 (derived from NF-κB2/p100). These proteins play important roles in regulating immune responses, inflammatory progress, and cell survival. Under normal resting conditions, NF-κB dimers binds IκB proteins, including IκBα, IκBβ, and IκBε, and are kept inactive in the cytoplasm. There are two main ways of signal transduction. In the canonical pathway, the IKK complex is activated by pro-inflammatory cytokines such as TNF-α and IL-1β, or pattern recognition receptors like TLR4. IKKα, IKKβ, and IKKγ are three subunits the complex contains. IKKβ phosphorylates IκBα specifically and marks it for ubiquitination and further proteasome degradation. Once IκBα is out of inhibition, the p65/p50 moves into the nucleus and turns on inflammation genes [5,16,19,22,29]. While the non-canonical pathway is triggered by specific receptors, including lymphotoxin-β receptor, CD40, BAFF-R, it depends on NF-κB-inducing kinase (NIK) and IKKα homodimers, which is different from the canonical pathway. The inactive p100 protein is conversed into p52, the active form of p100. Then, the formed RelB/p52 protein can turn on specified genes to induce chronic inflammation [5,16,30]. The canonical NF-κB pathway has sufficient evidence from countless in vitro and in vivo animal studies as the primary NF-κB subtype mediating CVD pathogenesis, while the non-canonical pathway has limited evidence in CVDs.

### 2.1. Relationship Between the Canonical NF-κB Pathway and CVDs

**Figure 1:**
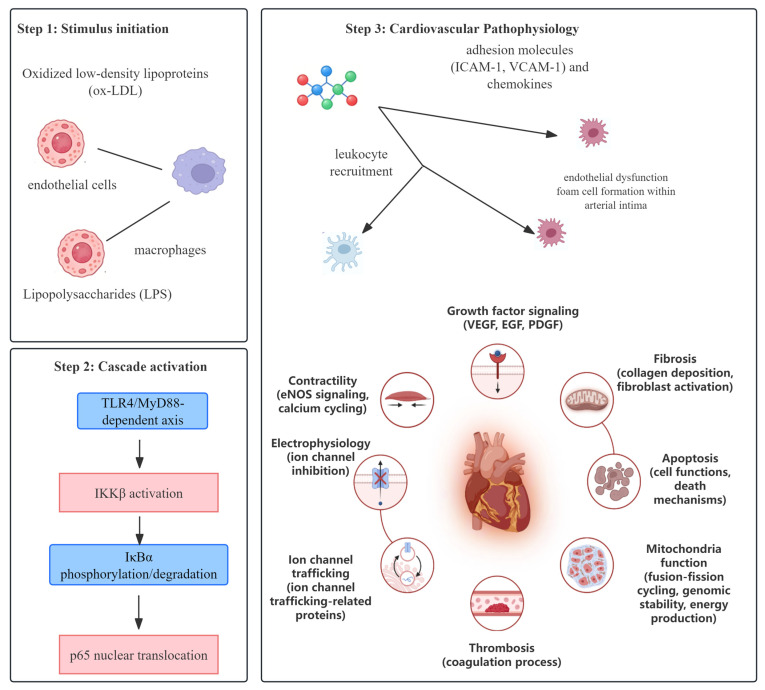
The relationship of canonical NF-κB signaling pathway and cardiovascular diseases. This figure shows three steps about the relationship between the canonical NF-κB pathway and cardiovascular diseases. First, oxidized low-density lipoproteins (ox-LDLs) and lipopolysaccharides (LPSs) affect endothelial cells and macrophages to start signaling. Then, the TLR4/MyD88-dependent axis transmits signaling, activates IKKβ, phosphorylates and degrades IκBα. p65 nuclear translocation happens. Activated NF-κB boosts adhesion molecules and chemokines. They drive immune cell recruitment, vessel lining damage, and artery clogging. These are the key steps in heart disease development.

The relationship between the canonical NF-κB signaling pathway and cardiovascular diseases is shown in Figure 1. The canonical NF-κB pathway is characterized by IKKβ-mediated IκBα phosphorylation/degradation and subsequent p65 nuclear translocation. It is the key circle in cardiovascular diseases, especially atherosclerosis. Atherosclerosis has the most sufficient evidence for canonical NF-κB involvement, with multiple independent in vitro (endothelial cell, macrophage) and in vivo (ApoE-/-, LDLR-/- mice) studies confirming its role in endothelial dysfunction, foam cell formation, and plaque development [8,10]. It induces endothelial dysfunction, macrophage activation, and foam cell formation and, finally, causes plaque formation in atherosclerosis [8,10]. Myocardial I/R injury has moderate to sufficient evidence for canonical NF-κB activation, with consistent findings across rodent models showing p65 nuclear translocation drives inflammatory cytokine release (TNF-α, IL-1β) and cardiomyocyte apoptosis [11,31]. NF-κB activation induces cardiac dysfunction and tissue damage by increasing TNF-α, IL-1β, IL-6, caspase-3 and Bax after myocardial ischemia-reperfusion; in cerebral ischemia-reperfusion, NF-κB has moderate evidence for enhancing oxidative stress and neuronal cell death, with studies showing consistent but less reproducible effects compared to cardiac I/R injury [31,32]. NF-κB activates neuroinflammation and oxidative stress, elevates blood pressure and damages cardiac function. Hypertension has moderate evidence for canonical NF-κB involvement in hypothalamic and vascular inflammation, with SHR models showing consistent effects but limited validation in other hypertensive rodent strains [7]. Upregulation of NF-κB induces inflammation and apoptosis in cardiotoxicity and then induces cardiac tissue damage by increasing inflammatory cytokines and caspase-3 [7,33]. Other studies have reported similar findings. Once NF-κB is activated, the p65 subunit moves from the cytoplasm into the nucleus and induces transcription of inflammatory genes. It can lead to cardiomyocyte apoptosis and blood–brain barrier injury. The key proteins contain TLR4, IκB, NLRP3 and inflammatory cytokines. The inflammatory cytokines also activate NF-κB. They form a vicious circle and speed the disease progress [34,35,36]. Across all CVDs, the TLR4/MyD88 upstream axis of canonical NF-κB has the most robust evidence, with its activation validated in nearly all CVD models and conserved across in vitro and in vivo systems [34,35].

### 2.2. Relationship Between the Non-Canonical NF-κB Pathway and CVDs

**Figure 2:**
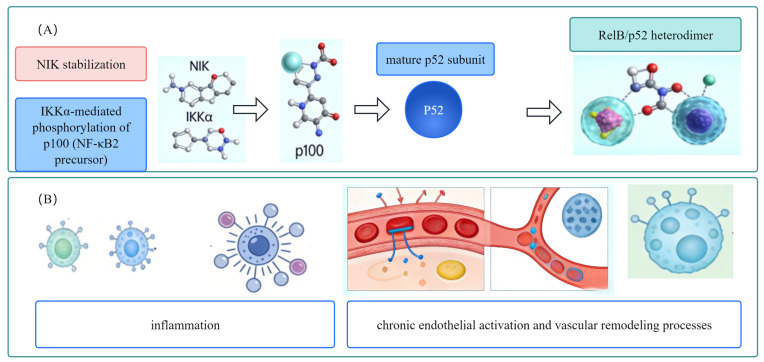
The relationship between the non-canonical NF-κB pathway and cardiovascular diseases. (**A**) Key signaling steps in the non-canonical NF-κB pathway: NIK stabilization promotes the phosphorylation of the p100 precursor, which is mediated by IKKα. Then, it leads to p100 processing into the mature p52 subunit. p52 with RelB forms a heterodimer and does downstream transcription. (**B**) The results of pathway activation are to induce inflammation and promote chronic endothelial activation and vascular remodeling. This will cause cardiovascular diseases.

The relationship between the non-canonical NF-κB pathway and cardiovascular diseases is shown in Figure 2. Non-canonical NFκB signaling is very important for hematopoietic development, secondary immune organs, and immune homeostasis. The RelB/p52 heterodimer regulates the genes expression about B-cell survival, lymphoid organogenesis, and sustained low-grade inflammation. The molecular network has shown that non-canonical NF-κB signaling modulates not only RelB but also the NF-κB subunits RelA and cRel [37]. We know less about the non-canonical RelB/p52 in CVDs than the canonical pathway. But it sustains inflammation in atherosclerotic plaques. Non-canonical NF-κB has limited evidence in CVDs: only a small number of studies link it to chronic atherosclerotic plaque inflammation, with no consistent findings in other CVD indications (e.g., myocardial I/R injury, hypertension, PAH) [37]. Blocking the NIK-IKKα-p100/p52 helps treat chronic inflammatory heart diseases, with this effect validated in only a single ApoE-/- mouse model of atherosclerosis, representing limited and unconfirmed evidence [37].

### 2.3. Crosstalk Between NF-κB and Other Pathways in CVDs

**Figure 3:**
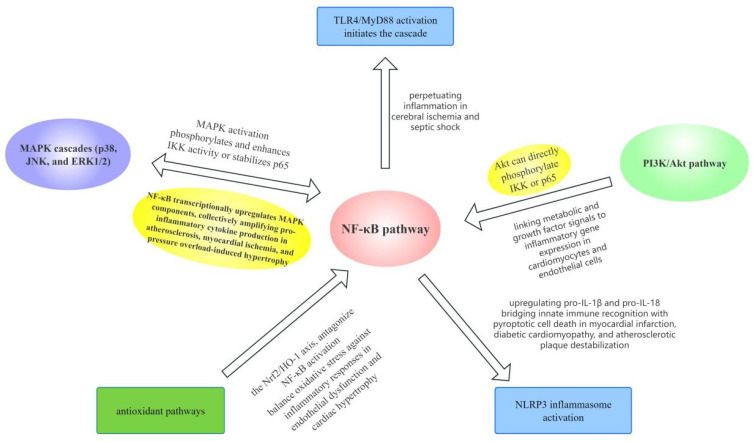
Crosstalk between NF-κB and other pathways in cardiovascular diseases. Figure 3 provides a picture about how NF-κB interacts with other pathways in cardiovascular diseases. NF-κB plays a key role in regulating inflammatory responses. MAPKs enhances the activity of IKK and the stability of p65. NF-κB upregulates the expression of MAPK components. The PI3K/Akt pathway directly phosphorylates IKK and p65. TLR4/MyD88 serves to initiate NF-κB activation.

The NF-κB signaling pathway does not work alone in cardiovascular diseases. Instead, it forms crosstalk with other pathways. This induces pathological processes, including inflammation, fibrosis, endothelial dysfunction and cardiac remodeling. The crosstalk between NF-κB and other pathways in cardiovascular diseases is shown in Figure 3. The crosstalk between NF-κB and MAPK/PI3K/Akt/TLR4 pathways has sufficient evidence across all major CVDs, with consistent mechanistic validation in both in vitro and in vivo studies [32,38,39]. In contrast, crosstalk with Nrf2/HO-1 and NLRP3 inflammasome has moderate evidence, with robust findings in atherosclerosis and myocardial I/R injury but limited validation in PAH and diabetic cardiomyopathy [29,36].

For example, MAPK is the upstream way and phosphorylates the IKK complex to accomplish NF-κB nuclear translocation. MAP4K4 is a key node, which links metabolic stress to vascular inflammation. MAPK-NF-κB crosstalk has sufficient evidence in atherosclerosis and myocardial hypertrophy, with multiple in vitro and animal studies confirming MAP4K4 as a critical mediator [32,38,39]. Their interactions induce pro-inflammatory cytokine release, leading to atherosclerotic plaque formation and myocardial hypertrophy [32,38,39]. Similarly, PI3K/Akt is another pathway and can either suppress or promote the NF-κB pathway in different cellular contexts. PI3K/Akt-NF-κB crosstalk has moderate evidence, with context-dependent effects (pro-inflammatory in hypertension, anti-inflammatory in cerebral I/R injury) validated in rodent models but with inconsistent findings across cell types [7,31]. PI3K/Akt can drive NF-κB inflammation and cause hypertension; PI3K/Akt phosphorylation activates NF-κB and inhibits the microglia phenotype from the M1 to M2 phenotype in cerebral ischemia/reperfusion injury [7,31]. Additionally, TLR4/NF-κB crosstalk has sufficient evidence in all CVDs, with TLR4 as the most well-validated upstream activator of canonical NF-κB in cardiac and vascular pathology [34,35]. TLR4 signaling is an important upstream activator of NF-κB in cardiac and vascular pathology. TLR4 turns on MyD88-dependent cascades, phosphorylates IKK and promotes NF-κB nuclear translocation. This progress induces inflammatory responses in cardiomyocytes and endothelial cells [34,35]. TLR4/NF-κB accelerates atherosclerosis through foam cell formation and plaque instability. Moreover, Src family kinases are also upstream factors and relate to NF-κB in vascular inflammation. Src-NF-κB crosstalk has limited evidence, with only a small number of studies linking it to endothelial barrier disruption in acute lung injury and no validation in other CVDs. Src phosphorylates AKT1, enhances IKK activity, and accomplishes NF-κB nuclear entry. It induces endothelial barrier disruption and leukocyte adhesion in acute lung injury [17]. The Nrf2/HO-1 antioxidant pathway exhibits differences. Nrf2/HO-1-NF-κB crosstalk has moderate evidence, with robust findings in atherosclerosis and myocardial I/R injury showing Nrf2 activation antagonizes NF-κB, but limited studies in PAH and diabetic cardiomyopathy. When Nrf2 signaling is damaged, NF-κB exhibits oxidative stress and neuroinflammation and accelerates tissue damage [29]. Furthermore, NF-κB-NLRP3 crosstalk has moderate to sufficient evidence in atherosclerosis and myocardial I/R injury, with p65 directly regulating NLRP3 expression validated in multiple models, but limited evidence in hypertension and PAH. NF-κB is the primary regulatory factor of the NLRP3 inflammasome and plays an important role in neurovascular inflammation. After nuclear translocation, p65 directly upregulates NLRP3 expression, and NLRP3 drives caspase-1 maturation and IL-1β/IL-18 release. This worsens and neuronal damage [36]. These examples confirm that the NF-κB pathway does not exist alone but exists in signaling network with upstream and downstream signaling, which induce cardiovascular pathologies, with the strength of evidence for each crosstalk axis varying by CVD indication and requiring further validation in understudied subtypes.

## 3. Cardiovascular Diseases and Pathological Roles

### 3.1. Atherosclerosis

The features of atherosclerosis are lipid deposition and plaque formation, which can narrow vessels. Atherosclerosis is the CVD indication with the most sufficient evidence for NF-κB-mediated pathogenesis, with cumulative data from hundreds of in vitro and in vivo studies confirming the role of NF-κB in all stages of plaque development (initiation, progression, instability) [3,10]. NF-κB activation, NLRP3 inflammasome release, and VCAM-1 expression induce inflammation and oxidative damage. Nrf2/HO-1 exhibits plaque protection. These cause pathology in the form of foam cells. During the progress, macrophage uptake of oxLDL and ROS drives it [8,10,40,41]. NOTCH1 signaling also participates the progress, and CD86, various scavenger receptors and NLRP3 induce oxidative damage and inflammatory responses. This leads to microvascular barrier dysfunction and leukocyte infiltration, which further causes tissue injury [17,27]. NF-κB, MAPK and NOTCH1 all regulate atherosclerosis. NF-κB has sufficient evidence as a core regulator, while MAPK has moderate to sufficient evidence, and NOTCH1 has limited evidence in atherosclerotic pathogenesis [3,10,27]. NLRP3, CD36, MMP-2/9, and Piezo1 regulate inflammatory responses, extracellular matrix degradation, and vascular remodeling, with NLRP3 and CD36 having sufficient evidence and MMP-2/9 and Piezo1 having moderate evidence as NF-κB downstream effectors in atherosclerosis [3,10].

### 3.2. Ischemic Cardiocerebrovascular and Organ Injury

NF-κB signaling plays a key role in ischemic injury of the heart, brain, and lungs. Myocardial I/R injury has moderate to sufficient evidence for NF-κB involvement, with consistent findings across rodent models and in vitro cardiomyocyte studies [11,42]. The specified characteristics of the MI/RI are oxidative stress, apoptosis, and inflammation. In MI/RI, p65 nuclear translocation, STAT3 activation and HMGB-1 release induce oxidative stress and cardiomyocyte injury, with STAT3 and HMGB-1 having moderate evidence as NF-κB-associated mediators in MI/RI [11]. In PIR, TLR4-MD-2-mediated NF-κB p65 phosphorylation also induces inflammation and causes tissue damage [42]. Even in myocardial infarction without reperfusion, NF-κB is regulated by the upstream ESR1-PI3K-Akt pathway. It activates the NLRP3 inflammasome to enhance inflammatory infiltration of myocardial tissue and cardiomyocyte apoptosis, with ESR1-PI3K-Akt having limited evidence as an NF-κB upstream pathway in permanent myocardial infarction [43]. This indicates that there are different upstream factors, like TLR4/MyD88 in the brain and lung, STAT3/HMGB-1 in the reperfused heart, and PI3K-Akt in permanent infarction. But NF-κB is the common factor among them, with sufficient evidence as a universal mediator of ischemic injury across cardiac, cerebral, and pulmonary tissues, despite tissue-specific upstream signaling axes with varying evidence strength.

### 3.3. Hypertension-Related Diseases

NF-κB plays a key role in hypertension pathogenesis. It is activated in the cardiovascular and brain regions and induces neuroinflammation and oxidative stress. The two aspects are the characteristics of hypertension disease. Hypertension has moderate evidence for NF-κB involvement, with consistent findings in SHR models but limited validation in other hypertensive rodent strains. The increase in NF-κB activity upregulates pro-inflammatory cytokines and NAD(P)H oxidase subunits and promotes ROS and sympathetic overactivation. The inflammation leads to vascular dysfunction and blood pressure elevation. NF-κB and other signaling pathways like MAPKs and PI3K/Akt together amplify neurohumoral excitation. MAPK-NF-κB and PI3K/Akt-NF-κB crosstalk have moderate evidence in hypertension, with consistent but not universally reproducible effects in hypothalamic and vascular tissue. These cause organ tissue damage, including cardiac hypertrophy and vascular remodeling [7,44]. In nitric oxide (NO)-deficient hypertension, sustained blood pressure elevation, left ventricular hypertrophy, and vascular dysfunction are its characterization. Lack of NO shortage for a long time leads to free radicals accumulation, inflammation, and NF-κB activation. NF-κB induces production of inflammatory substances like TNF-α and TGF-β1, while TNF-α stimulates NF-κB activity by TNFR1 and TNFR2 [24]. The hypertensive disorder of pregnancy is characterized by endothelial dysfunction, inflammation, and oxidative stress. Preeclampsia has limited evidence for NF-κB involvement, with findings from in vitro endothelial cell and placental explant studies. Pro-inflammatory cytokines promote NF-κB nuclear translocation and ROS, ET-1, and IL-6 production in preeclampsia. These factors lead to vasoconstriction and vascular inflammation, which clearly create the maternal syndrome. Placental hypoxia and ischemia go on amplifying NF-κB signaling and exacerbate pathological changes [18]. PAH has moderate evidence for NF-κB involvement in vascular remodeling and right ventricular dysfunction, with consistent findings in monocrotaline and hypoxic rodent models but limited mechanistic depth. NF-κB signaling regulates vascular inflammation and remodeling in PAH [12,45]. NF-κB with long time activation causes endothelial dysfunction, smooth muscle cell proliferation, extracellular matrix deposition, obliterative vascular lesions and right heart failure [12]. It induces IL-6 and IL-1β production, enhances pyroptosis through NLRP3 inflammasome signaling crosstalk, and accelerates muscularization of distal arteries by linking TGF-β signaling, with NLRP3 and TGF-β having limited evidence as NF-κB downstream effectors in PAH [12,45]. The activation of NF-κB/NLRP3 under hypoxic conditions directly promotes pyroptosis in pulmonary arterial smooth muscle cells, exacerbates pulmonary vascular remodeling, and induces pulmonary hypertension [45].

### 3.4. Other CVDs

Cardiac hypertrophy is another CVD with a characterization of abnormal myocardial thickening and impaired cardiac function. NF-κB leads to these results after stimulation by ROS and upstream kinases. Pro-inflammatory cytokines are the weapon of NF-κB and induce myocardial inflammation and hypertrophy [44,46,47]. The expression of ANP/BNP increases [46,47]. SIRT1 dysfunction activates NF-κB, while MAPKs (p38, ERK1/2) and AKT phosphorylation promotes the inflammatory effects of NF-κB, with SIRT1 having moderate evidence and MAPK/AKT having sufficient evidence as NF-κB-associated regulators in cardiac hypertrophy [44,46]. ROS/NO imbalance makes NF-κB stay in the nucleus and go on inducing inflammation [44,47]. The structure of the heart cells gets disrupted, energy supply breaks down, and the heart slowly loses its ability to pump blood [46,47].

Cardiomyopathies (toxic, septic, diabetic) have moderate evidence for NF-κB involvement overall: septic cardiomyopathy has sufficient evidence with consistent findings in rodent models [19,48], diabetic cardiomyopathy (DCM) has moderate evidence with robust but less reproducible findings [13,21], and toxic cardiomyopathy has moderate evidence with model-specific effects [21,33]. NF-κB activation induces myocardial inflammation, oxidative damage, and apoptotic cell death. Cardiac dysfunction, fibrotic deposition, and chamber dilation are their characterizations. There are different kinds of cardiomyopathies, including toxic, septic, and diabetic [21,48,49]. NF-κB is left in the nucleus by TLR4, ROS produced by NOX4, and abnormal FOXO1, with TLR4 having sufficient evidence in septic cardiomyopathy and moderate evidence in DCM, and NOX4/FOXO1 having limited evidence in toxic/DCM [21,48]. The activation of ERK1/2 promotes NF-κB to work hard with serious inflammatory responses [21]. The vicious circle goes on and finally leads to mitochondrial dysfunction, contractile protein degradation, and eventual pump failure [21,49].

Vascular calcification and aortic valve disease have limited evidence for NF-κB involvement, with only a small number of in vitro and animal studies validating its role in osteogenic differentiation of vascular and valvular interstitial cells. Persistent NF-κB activation breaks nitric oxide bioavailability, promotes vascular smooth muscle proliferation, and accelerates matrix deposition. It presents arterial stiffness, intimal thickening, and flow-mediated dilation damage [15]. AGE-RAGE signaling and ROS make NF-κB stay in the nucleus. The activation of ERK1/2 also promotes NF-κB to induce inflammation of vascular tissues and finally leads to endothelial dysfunction, calcific remodeling, and luminal compromise [15,50]. NF-κB can transform quiescent valvular interstitial cells into osteoblast-like phenotypes. Runx2, BMP2 and osteopontin are upregulated and cause leaflet thickening, stiffness, and obstructive outflow gradients. AKT and ERK1/2 cascades amplify the function of NF-κB. Meanwhile, ROS produced by NOX2/p22phox promotes the pro-calcific milieu. These together ultimately cause progressive valve obstruction, left ventricular pressure overload, and eventual pump failure, with Runx2/BMP2 having limited evidence as NF-κB downstream effectors in aortic valve disease [50].

## 4. Types and Mechanisms of Natural Compounds in the Treatment of Cardiovascular Diseases

Natural products have a variety of advantages to treat CVDs, including their structural diversity, low toxicity, and multi-target biological activities. According to their chemical structures, the categories of natural products mainly include flavonoids, terpenoids, phenylpropanoids, alkaloids, quinones, steroids, polyphenols, saccharides, tannins, and other categories. Traditional Chinese medicine (TCM) compound prescriptions exert effects on CVDs treatment through complex compounds. They all exhibit cardioprotective effects via the NF-κB signaling pathway.

**Figure 4:**
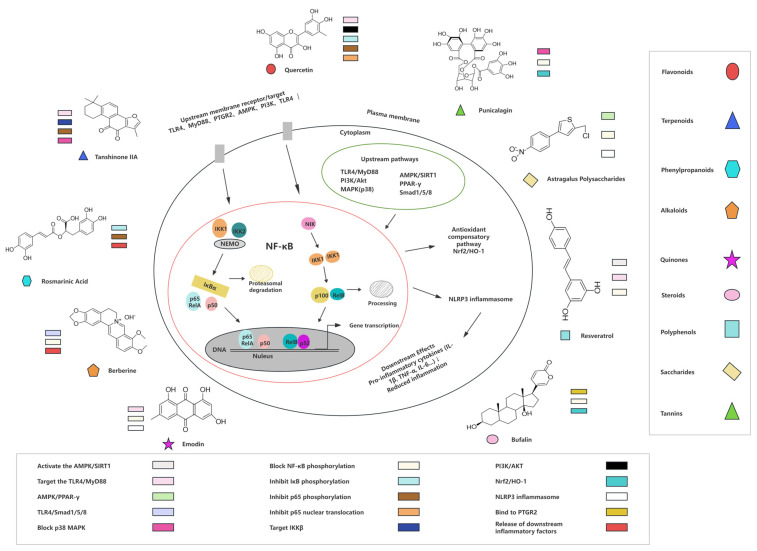
Chemical structure of natural products and their action sites on NF-κB signaling pathway.

This section provides a critical, evidence-based analysis: for each natural product class and individual compound, we analyze evidence based on the number, type, and reproducibility of studies (in vitro, in vivo animal, human clinical), evaluate efficacy by specific CVD indication, and highlight the most validated compounds and their translational potential. NF-κB signaling participates in pathological processes of CVDs such as atherosclerosis, myocardial infarction, hypertension, and heart failure. Pro-inflammatory cytokines (TNF-α, IL-1β) and oxidative stress stimulate the formation of the IκB kinase (IKK) complex, induce phosphorylation and degradation of IκB, and release NF-κB dimers (predominantly p65/p50) to translocate into the nucleus, finally inducing the gene expression of pro-inflammatory factors, adhesion molecules, and matrix metalloproteinases. Because of the effects of NF-κB in CVDs, more researchers focus on natural products and hope to find a viable way to treat CVDs. We summarize the types and mechanisms of natural products targeting the NF-κB pathway to treat CVDs; see Table 1 and Figure 4. This also includes study type (in vitro/in vivo/human) for each compound and its associated CVDs indication, enabling direct comparison of evidence strength across compounds and indications. The following is a detailed description according to the chemical types, with critical analysis and emphasis on evidence.

Figure 4 illustrates the basic chemical structure and a few representative structures of natural compounds. Each category of natural products (e.g., flavonoids, terpenoids, alkaloids) is mapped to its experimentally validated target sites reported in published studies. For instance, flavonoids, like quercetin, act on IκB phosphorylation, p65 nuclear translocation, and upstream TLR4/MyD88 and PI3K/Akt pathways, while associating with the Nrf2/HO-1 pathway. The diagram also annotates the regulatory crosstalk between NF-κB and intersecting pathways such as Nrf2/HO-1, SIRT1, and NLRP3. Representative compounds with the most extensive research evidence in each class are selected to ensure the credibility of the target interactions.

### 4.1. Flavonoids

Flavonoids are characterized by a C6-C3-C6 structure, belonging to polyphenolic compounds. Flavonoids are a kind of secondary metabolite produced by plants. In medicinal plants, both free flavonoids and flavonoid glycosides exhibit a wide range of physiological functions and hold great application value. They become effective regulators of the NF-κB signaling pathway, and provide a promising treatment strategy for CVDs, which are characterized by chronic inflammation and oxidative stress. Flavonoids are the most well-studied natural product class for NF-κB-mediated CVDs treatment, with overall moderate to sufficient evidence across multiple CVDs indications; atherosclerosis and myocardial I/R injury are the most validated, while PAH and preeclampsia have moderate to limited evidence [7,8,10,21]. They can regulate NF-κB activity, and then reduce vascular remodeling, myocardial injury, and endothelial dysfunction.

Atherosclerosis is a common cardiovascular disease. The main pathological change is plaque formation on the arterial intima, including fatty streaks to fibrous plaques and ultimately atheromatous plaques. Kaempferol has sufficient evidence for atherosclerosis treatment, with multiple ApoE-/- mouse models and in vitro macrophage studies confirming its NF-κB inhibitory effects and anti-foam cell formation [10,51]. It also has moderate evidence for NO-deficient hypertension [24]. Kaempferol significantly reduces atherosclerotic plaque formation in mice. In vitro experiments show that kaempferol inhibits macrophage foaming by regulating the MAPK/NF-κB and Nrf2/HO-1 pathways. During the procedure, inflammatory response, CD36 expression, mitochondrial membrane potential elevation, ROS production, MAPK/NF-κB expression and Ca^2+^ influx are all inhibited. However, Nrf2/HO-1 levels are promoted [10]. Additionally, kaempferol can protect against cardiovascular abnormalities induced by nitric oxide deficiency in rats. Kaempferol lowers blood pressure and improves cardiovascular malfunction and provoked hypertrophy. The TNF-α pathway plays a key role in its progress. Kaempferol inhibits the expression of tumor necrosis factor receptor 2, PI3K, Akt1, and smad2/3 in heart tissue, while it upregulates tumor necrosis factor receptor 1 (TNFR1), phosphorylated nuclear factor-kappaB (p-NF-κB), and transforming growth factor beta 1 (TGF-β1) in vascular tissue [24]. Kaempferol biomimetic nanoparticles (KPF@MM-NPs) have moderate evidence for enhanced anti-atherosclerotic efficacy, with a single ApoE-/- mouse study showing targeted M1-to-M2 macrophage polarization. It is a delivery system coated with macrophage membrane designed for kaempferol. The aim was to enhance the clinical efficacy of anti-inflammatory drugs to treat atherosclerosis and its emergence of complications. This was achieved through transforming pro-inflammatory M1 macrophages into an anti-inflammatory M2 phenotype in atherosclerotic ApoE-/- mice. During this period, the ROS/NF-κB signaling pathway is blocked, while the production of pro-inflammatory cytokines is decreased [51]. Kaempferol-3-O-rutinoside has higher water solubility than kaempferol. It is a flavonoid glycoside from Lu’an GuaPian tea and prevents and treats Ventricular remodeling (VR) after acute myocardial infarction (AMI). But it has limited evidence for ventricular remodeling post-AMI, besides decreasing the expressions of NF-κB p65, NLRP3, ASC, Caspase-1, GSDMD and IL-1β [70].

Morin hydrate has moderate evidence for atherosclerosis and endothelial inflammation, with consistent findings in LPS-induced HUVECs and ApoE-/- mice. In LPS-induced human umbilical vein endothelial cells, morin hydrate inhibits monocyte adhesion, the expression of intercellular adhesion molecule-1, vascular cell adhesion molecule-1, cyclooxygenase-2, and matrix metallopeptidase 9, and induces autophagy via inhibiting the PI3K/Akt/NF-κB pathway [57]. (2S)-5-methoxy-6-methyl-flavan-7-ol from *Sanguis draconis* reduces atherosclerosis by inhibiting the NF-κB/IκBα pathway, downregulating intercellular adhesion molecule-1 and reducing monocyte–endothelial adhesion [59]. It needs more evidence for atherosclerosis treatment.

Apigenin has sufficient evidence for atherosclerosis, with multiple ApoE-/- mouse models and in vitro macrophage studies confirming its NF-κB p65 nuclear translocation inhibition and anti-pyroptosis effects. It can inhibit macrophage pyroptosis and atherosclerosis. It reduces oxidative stress, blocks NF-κB p65 nuclear translocation, and inhibits NLRP3 inflammasome activation of the plaque [8].

Isoliquiritigenin (ISL) is a bioactive compound isolated from licorice. It has been shown to reduce neuroinflammation following subarachnoid hemorrhage (SAH). In the SAH rat model, ISL inhibits NLRP3 inflammasome activation by blocking NF-κB, inhibiting NF-κB p65 translocation. Then, it inhibits the expression of pro-inflammatory cytokines, including TNF-α, IL-6, IL-1β, and IL-18. It also reduces brain edema and maintains blood–brain barrier integrity [36]. It shows limited evidence for subarachnoid hemorrhage (SAH)-induced neuroinflammation, without multi-studies validation.

Luteolin has sufficient evidence for essential hypertension [7] and limited evidence for preeclampsia [18]. Luteolin is widely distributed in nature. It was firstly isolated from the leaves, stems, and branches of *Reseda odorata* L., which belongs to the genus Reseda in the family Resedaceae. Now, luteolin has been found in natural medicinal herbs such as *Dracocephalum integrifolium*, *Lonicera japonica*, and *Perilla frutescens*. It exhibits positive effects in regulating hypertension. The hypothalamic paraventricular nucleus is a critical central regulator of blood pressure. Luteolin can inhibit inflammatory responses and oxidative stress mediated by the PI3K/Akt/NF-κB signaling pathway in the hypothalamic paraventricular nucleus of spontaneously hypertensive rats (SHRs). By lowering the levels of associated inflammatory cytokines and oxidative products, luteolin exerts a marked hypotensive effect, thereby expanding our understanding of the mechanisms underlying luteolin-mediated blood pressure regulation [7]. Preeclampsia (PE) is another kind of hypertension which is a serious hypertensive pregnancy disorder with inflammation, oxidative stress, and angiogenic imbalance. Luteolin significantly reduces NF-κB activation, reactive oxygen species, superoxide, and IL-6 and ET-1 expression in endothelial cells, while significantly reducing the phosphorylation of NF-κB in human placental explants [18].

Galangin reduces cardiac injury, balances redox, and upregulates SIRT1, Nrf2, SIRT3, and mitochondrial transcription factor to improve cyclophosphamide-induced cardiotoxicity of rats [56]. It validates its NF-κB inhibitory and mitochondrial protective effects in animal models.

Quercetin has moderate evidence across multiple CVDs. These include cardiotoxicity [21,33], cerebral I/R injury [31], PAH [52], and intimal hyperplasia [26]. It shows consistent NF-κB inhibitory effects. However, its clinical translation is limited due to poor bioavailability. Its nanosuspension (Qu-PEG NS) has good evidence for improved bioavailability and efficacy [53]. Quercetin exhibits many cardioprotective effects, including cardiotoxicity [21,33], intimal hyperplasia post-balloon injury [26], cerebral ischemia/reperfusion injury [31], and pulmonary arterial hypertension [52]. Quercetin exhibits significant protection against aluminum phosphide or 5-fluorouracil. It owes its cardiac tissue protection to its antioxidant, anti-inflammatory, and anti-apoptotic properties. Multiple signaling pathways, NOX4, FOXO1, ERK1/2, NF-κB, and Nrf2, are regulated by quercetin [21,33]. Using the cerebral ischemia/reperfusion injury rat model, quercetin ameliorates brain damage and regulates the related marker expression of microglia/macrophage M1/M2 polarization. It is regulated through the PI3K/Akt/NF-κB signaling pathway [31]. The excessive proliferation of neointimal tissue is the characterization of percutaneous coronary interventions for vascular restenosis. Quercetin, paired with exosomes, reduces neointimal hyperplasia by inhibiting cell proliferation in a Sprague Dawley rat model. It does not depend on the lipoxygenase but on Akt, NF-κB, and ERK signaling pathways to regulate cell proliferation [26]. Moreover, quercetin also improves pulmonary arterial hypertension in rats through HMGB1/RAGE/NF-κB pathway regulation [52]. But quercetin has low bioavailability because of its poor water solubility, low absorption, and rapid excretion in the body. A Qu and polyethylene glycol nanosuspension (Qu-PEG NS) is developed and shows comparable antioxidant, anti-inflammatory, and antibacterial functions with safety, good solubility, and physical stability [53].

Baicalin is isolated from *Scutellaria baicalensis* and exhibits cardioprotective effects through multiple NF-κB related mechanisms. It inhibits thrombus formation in deep vein thrombosis model rats and enhances the migratory and angiogenetic abilities of endothelial progenitor cells by regulating upregulating SIRT1 and suppressing NF-κB signaling [54]. Similarly, baicalin-pretreated mesenchymal stem cell-derived exosomes (Ba-exos) inhibit atherosclerotic plaque formation in mice [25]. In addition, baicalin reduces doxorubicin-induced cardiotoxicity and inhibits cardiac fibroblast activation in obesity-related cardiac fibrosis via inhibiting the NF-κB pathway [34,55]. It has sufficient evidence for atherosclerosis and moderate evidence for cardiotoxicity, deep vein thrombosis, and cardiac fibrosis. Its NF-κB inhibitory mechanism is well-validated.

Scutellarin is another flavonoid from *Scutellaria baicalensis*. It shows protective effects for cerebral ischemia/reperfusion (I/R) injury. During the procedure, it inhibits NF-κB p65 and p38 MAPK signaling and reduces oxidative stress and inflammatory responses [32]. Wogonoside is also from *Scutellaria baicalensis* and reduces atherosclerosis in ApoE-/- mice. Wogonoside reduces aortic inflammatory responses, lipid deposition, and pro-inflammatory cytokine release by inhibiting the TLR4/NF-κB pathway [35].

Liquiritin is a flavonoid glycoside isolated from licorice. It shows protective effects of myocardial fibrosis after myocardial infarction (MI). It significantly inhibits collagen I, collagen III, TGF-β1, MMP-9, α-SMA, CCL5, and p-NF-κB [67]. Liquiritin decreases the levels of ROS, malonedialdehyde (MDA), lactate dehydrogenase (LDH), TNF-α, IL-1β and IL-6 and increases ATP, SOD, GSH-px, glutathione reductase (GR) and catalase (CAT) activities in oxidative damage of H9c2 cardiomyocytes [68]. Liquiritin exerts cardioprotective effects via NF-κB-related pathways [67,68], with sufficient evidence from rat MI models and moderate evidence from H9c2 cell experiments.

Phloridzin [75] has moderate evidence (animal/H9C2 models) against diabetic cardiomyopathy (DCM) and lacks integrated evidence comparison. It decreases the levels of myocardial injury markers of DCM, such as lactate dehydrogenase and creatine phosphokinase-MB. It also inhibits myocardial collagen fiber accumulation and myocardial inflammation by inhibiting the MyD88/NF-κB signaling pathway [75].

Astilbin [76] shows sufficient in vivo/in vitro evidence for sepsis-induced cardiac injury. It reduces sepsis-induced cardiac injury by inhibiting the TLR4/NF-κB pathway. It activates the Nrf2/HO-1 pathway, reducing myocardial inflammation, oxidative stress, and apoptosis and improving cardiac function and electrical remodeling [76].

By inhibiting NF-κB/MAPK signaling, fisetin also reduces cardiac inflammation, myocardial hypertrophy, and the fibrosis of cardiomyopathy. During the procedure, fisetin also inhibits the MAPK pathway [38]. It has sufficient in vivo and in vitro evidence for obesity cardiomyopathy via NF-κB/MAPK inhibition. Alpinetin reduces cardiac inflammation and remodeling post-acute myocardial infarction via the TLR4/MyD88/NF-κB pathway [58]. It shows moderate evidence for ischemic heart disease.

Naringin (NG) treats radiation-induced heart disease (RIHD) by reducing cardiac dysfunction, fibrosis, inflammation, and oxidative stress and inhibiting endoplasmic reticulum stress. In terms of mechanism of action, NG activates the Sirt1 signaling pathway and inhibits NF-κB phosphorylation. Then, it blocks downstream inflammatory and fibrotic responses [74]. Naringin has limited evidence for RIHD and needs further study.

Breviscapine has protective effects for cerebral ischemia-reperfusion injury but with limited evidence. These effects include reducing the neurological injury, infarct volume and serum NSE concentration of rats. It regulates intestinal flora homeostasis, inhibits the TLR4/MyD88/NF-κB inflammatory pathway in the brain, and regulates CYP3A4 expression in different intestinal segments of rats [66].

Neuroinflammation is not only a trait of neurological disorders but also a key mechanism underlying the development and progression of CVDs. Amentoflavone shows anti-neuroinflammation and oxidative stress by inhibiting TLR4/MyD88/NF-κB and activating Nrf2/HO-1 pathways [29]. It shows limited evidence restricted to in vitro BV2 microglia experiments for neuroinflammation.

Hydroxysafflor yellow A (HSYA) from *Carthamus tinctorius* shows sufficient evidence from both in vitro BV2 cells and in vivo mouse MCAO/R models of cerebral I/R injury. HSYA has a protective effect for cerebral ischemia and can reduce neuroinflammation in cerebral ischemia-reperfusion injury by making microglia polarization transform the pro-inflammatory M1 phenotype into the anti-inflammatory M2 phenotype. The procedure depends on activating SIRT1, enhancing its interaction with HMGB1, and further inhibiting the downstream NF-κB signaling pathway [20].

Icariside II has the effect of neuroprotection by reducing cerebral injury in the mouse model and activating astrocytic Nrf2. The OXPHOS/NF-κB/ferroptosis pathway is regulated by icariside II during the neuroprotection procedure [69]. It shows sufficient evidence from both in vitro astrocyte and in vivo mouse MCAO models against ischemic stroke.

Genistein-3′-sodium sulfonate (GSS) also exhibits a neuroprotective effect for cerebral ischemia-reperfusion injury (CI/RI). It promotes brain function recovery. The effect is realized by its inhibition of the TLR4/NF-κB pathway and further enhancing astrocyte polarization to the A2 phenotype [77]. Moderate evidence supports its effects in rat stroke models.

Many flavonoids exhibit their improvement effects on cardiovascular dysfunction by regulating the NF-κB signaling pathway, with the vast majority of supporting evidence derived from in vitro cell experiments and animal models, and limited human clinical trial data available to validate these effects to date. *Lycium ruthenicum* Murray anthocyanins (LRAs) improves aging-related cardiovascular dysfunction by reducing oxidative stress, inflammation, and apoptosis via the SIRT1/P53 signaling axis [78], an effect only confirmed in *D*-galactose-induced H9c2 cell models and H2O2-treated zebrafish models. Dihydromyricetin protects vascular function under hyperglycemia conditions by inhibiting HIF-1α/ROR2/NF-κB signaling [60], with its protective role in hyperglycemic endothelial dysfunction supported by multiple functional and molecular assays in in vitro rat aortic ring models, providing moderate preclinical evidence. 7-Procyanidin B2 protects retinal microvascular cells from hyperglycemia-induced stress by regulating redoxosomes/NF-κB signaling [65], a finding restricted to in vitro rat retinal capillary endothelial cell models with limited evidence focused on diabetic retinopathy-associated microvascular dysfunction.

Epigallocatechin-3-gallate (EGCG), pelargonidin, and genistein exhibit significant protective effects against vascular endothelial injury and inflammation, mainly through regulating the NF-κB signaling pathway [62,63], and this core mechanism has been validated with moderate to sufficient preclinical evidence across distinct endothelial injury models. EGCG reduces ethanol-induced endothelial cell injury by regulating NF-κB translocation and activating Nrf2 [63]. Pelargonidin reduces vascular inflammation by decreasing the levels of PGE2, IL-1β, IL-6, IL-8 and TNF-α via inhibiting NF-κB and the expression of COX-2 [62], with its protective effect on human umbilical vein endothelial cells (HUVECs) supported by multiple concentration gradient experiments and positive comparisons with NF-κB pathway inhibitors. It shows sufficient in vitro evidence for this indication. Genistein is a CB1 antagonist and exhibits an inhibitory effect on marijuana-induced vascular inflammation [61]. Genistein has been studied comprehensively. Research includes in vitro human-induced pluripotent stem cell-derived endothelial cell models. It also includes in vivo mouse atherosclerosis models. Supplementary epidemiological correlation analysis was conducted. This provides sufficient preclinical evidence. It is one of the most comprehensively studied flavonoids for vascular inflammatory injury via the NF-κB pathway.

Linarin (LIN) reduces fenvalerate-induced cardiotoxicity by reducing pro-inflammatory cytokines, oxidative stress markers, and apoptosis. It regulates the TLR4-mediated HMGB1/RAGE/NF-κB signaling axis [71], an effect confirmed in Sprague Dawley rat in vivo models with moderate preclinical evidence from a single animal species study. Similarly, diosmin-loaded chitosan nanoparticles (DCNPs) significantly reduce doxorubicin-induced cardiotoxicity by improving oxidative imbalance and inhibiting the NF-κB and apoptosis [72]. The results highlight the potential of flavonoid derivatives or delivery systems to boost efficacy and absorption.

Hesperidin (HES) improves monocrotaline-induced pulmonary arterial hypertension (PAH). It inhibits the NF-κB pathway, reduces inflammatory responses and pulmonary vascular remodeling, and improves pathological conditions such as right ventricular hypertrophy [12], with this effect supported by multiple dose gradient experiments (20 mg/kg and 40 mg/kg) and comprehensive detection of hemodynamic, pathological, and molecular indicators in rat models, providing sufficient in vivo preclinical evidence for PAH treatment. It reduces Kawasaki disease (KD)-related vascular endothelial injury by inhibiting inflammation, ROS production and endothelial cell apoptosis. The procedure is realized by inhibiting the TLR4/IκBα/NF-κB pathway [73]. This finding was validated in both CAWS-induced mouse in vivo models and TNF-α-induced HUVEC in vitro models. It exhibits sufficient preclinical evidence for KD-associated vascular injury. It makes hesperidin one of the flavonoids with the most robust preclinical evidence for specific CVD subtypes via NF-κB regulation.

Norwogonin (NWG) can inhibit inflammation and modify the pulmonary microvascular barrier function of rats with deteriorated arterial blood gas caused by LPS [17]. This effect is validated in mouse AF models. It is supported by reverse verification with proteasome activators. It provides moderate to sufficient preclinical evidence. It fills a research gap for flavonoids in anti-arrhythmic therapy via the NF-κB pathway. Atrial fibrillation (AF) is the most common type of cardiac arrhythmia and a significant contributor to stroke occurrence. 4-Hydroxychalcone (4HCH) exhibits a protection effect in atrial fibrillation (AF) induced by angiotensin II, including reducing atrial inflammation, oxidative stress, and fibrosis, and decreasing the incidence and duration of angiotensin II-induced atrial fibrillation. It inhibits immunoproteasome activity and its catalytic subunits. After blocking the IKKα/β-NF-κB signaling pathway, it regulates the downstream pathway, together reducing AF [61].

In summary, many flavonoids, such as kaempferol, quercetin and baicalin, treat CVDs mainly by regulating the NF-κB signaling pathway, reducing inflammation, oxidative stress and tissue damage. So, they improve diseases like atherosclerosis, myocardial injury and hypertension. Evidence for these common CVDs is mostly scattered across individual preclinical studies, with no systematic comparative research to validate relative efficacy, and limited to in vitro and animal models with no human clinical confirmation. Some flavonoid derivatives or delivery systems also enhance their effectiveness and absorption. Though this potential is supported by preclinical data (e.g., DCNPs for cardiotoxicity), evidence for improved bioavailability and targeting is limited to single animal studies, with no optimized structural modification or formulation design tailored to the NF-κB pathway as a specific target.

### 4.2. Terpenoids

Terpenoids are formed by the condensation of isoprene units. They include monoterpenes, sesquiterpenes, diterpenes, volatile oils, triterpenes, tetraterpenes, triterpenoid saponins, etc. They modulate the NF-κB pathway through specified mechanisms to prevent and treat CVDs. Terpenoids target the NF-κB pathway through diverse upstream regulators and downstream effectors. The evidence supporting their efficacy varies widely. Some compounds have robust validation from multiple in vivo and in vitro studies. Others rely on limited data from single experiments. Importantly, human clinical evidence is almost entirely lacking for terpenoid applications in cardiovascular diseases. This gap represents a major barrier to clinical translation.

Sweroside and catalpol of monoterpenoids regulate the NF-κB signaling pathway and inhibit atherosclerosis by anti-inflammation and protection of endothelial cells. Sweroside has been confirmed in both palmitic acid-induced endothelial cell injury models and western diet-fed atherosclerotic mouse models to target the MAP4K4/NF-κB axis, reducing vascular inflammation, endothelial adhesion responses, leukocyte homing, and atherosclerotic lesion formation—evidence for this mechanism is sufficient, with consistent results across independent in vitro and in vivo experiments [39]. Catalpol, by contrast, inhibits hyperhomocysteinemia (HHcy)-induced endothelial mesenchymal transition (EndMT), a critical process in atherogenesis, via the ROS/NF-κB pathway. This effect is supported by medium evidence from both HHcy-treated human umbilical vein endothelial cells (HUVECs) and high-methionine-diet-fed mouse models, where catalpol reduces aortic endothelial ROS levels, reverses aortic root endothelial pathological changes, and downregulates α-SMA, N-cadherin, and p-p65 expression [79]. Rehmannioside A and paeoniflorin treat hypertensive nephropathy and pulmonary arterial hypertension, respectively, with medium evidence from rodent models. They inhibit inflammation and pulmonary arterial remodeling via the MAPK/NF-κB pathway [83,101]. Moreover, rehmannioside A increases the expression of angiotensin converting enzyme 2 (ACE2) and inhibits the expression of angiotensin II type 1 receptor (AT1R), ACE, IL-17, mitogen activated protein kinase 14 (MAPK14), phosphorylated (p)-MAPK14, p-NF-κB P65, and matrix metallopeptidase 9 (MMP-9) in hypertensive nephropathy mice or in rat tubular epithelial cells [80]. Paeoniflorin blocks endothelial–mesenchymal transition and inhibits phosphorylation of transforming growth factor-β activated kinase 1 (TAK1) [101]. Aucubin is an iridoid glycoside and has a protective effect in cardiomyocyte injury caused by H/R and myocardial injury caused by I/R. The effect is to inhibit oxidative stress, inflammation and apoptosis. The main mechanism is the STAT3/NF-κB/HMGB-1 pathway. Aucubin affects STAT3 phosphorylation and inhibits the nuclear translocation of NF-κB and the release of HMGB1 to protect the myocardium [11]. Cornel iridoid glycosides (CIG) is isolated from *Cornus officinalis* Sieb. et Zucc. and shows neuroprotective effects against cerebral ischemia/reperfusion injury. It reduces microglia aggregation, neuroinflammatory responses, and brain cell apoptosis by inhibiting the TLR4/MyD88/NF-κB pathway [97]. Evidence for this effect is medium, derived from transient middle cerebral artery occlusion/reperfusion (tMCAO/R) rat models where CIG dose-dependently reduces microglia aggregation, neuroinflammation, and brain cell apoptosis.

Sesquiterpenes act as modulators of the NF-κB signaling pathway to treat CVDs. Evidence strength varies widely across subclasses. Some areas have solid backing from multiple studies, particularly for atherosclerosis and cerebral ischemia-reperfusion injury. Other areas rely on just one experiment, such as drug-induced cardiotoxicity and myocardial infarction. Lactucopicrin comes from *Cichorium intybus* L. and belongs to the sesquiterpene lactone family. It shows strong anti-inflammatory and anti-atherosclerotic effects. Multiple studies back this up, using both LPS-stimulated macrophages and high-fat diet-fed ApoE-/- mice. The compound reduces plaque area and stops macrophage NF-κB activation. Interestingly, it works through a unique AHR-NF-κB crosstalk mechanism rather than impacting NF-κB directly [83,84]. Isolinderalactone (ILL) significantly reduces atherosclerotic lesion size, foam cell formation, and pro-inflammatory factor expression in HFD-fed ApoE-/- mice by inhibiting the NF-κB pathway [40]. Costunolide (CTD) also improves AS in HFD-fed ApoE-/- mice. It covalently binds to cysteine 179 on IKKβ, inhibits its phosphorylation and changes its active loop to achieve NF-κB inactivation [16]. Atractylenolide III (ATL III) significantly reduces infarct volume, neurological deficits, and pro-inflammatory cytokine release. It protects blood–brain barrier (BBB) integrity in MCAO mice. Mechanistic studies demonstrate that it is related to the PI3K/Akt/NF-κB pathway, due to reduce oxidative stress, apoptosis, and inflammatory mediator production [82]. Cedrol is isolated from ginger. It regulates the ERβ-NF-κB pathway, inhibits microglia-mediated neuroinflammation, reduces cerebral infarct volume and improves neurological function. It shows obviously neuroprotective effects against acute cerebral ischemic injury, with medium evidence from LPS-challenged microglia and MCAO mice [90]. Vernodalin (VN) has cardioprotective efficacy in isoproterenol-induced myocardial infarction (MI), including inhibiting histopathological changes, reducing heart weight/body weight index, and decreasing cardiotoxicity enzymes, biomarkers, and inflammatory cytokines. It inhibits inflammation by regulating the NF-κB, VEGF-B, AMPK, and eNOS signaling pathways. However, the evidence is limited, as it is derived from only single studies using isoproterenol-induced myocardial infarction (MI) in rats [85]. Valencene inhibits cardiac hypertrophy, oxidative stress, and the NF-κB inflammatory pathway to give rise to anti-inflammation and a minimum of myocardial infarct size [86]. The use of doxorubicin (DOX) causes cardiotoxicity with abnormal heart weight, serum cardiac biomarker levels, oxidative stress, and histopathological alterations. Santonin (Sant) and nerolidol (NERO) could reduce DOX-induced cardiotoxicity. Sant downregulates TLR4, NF-κB, and caspase-3, deceases the levels of pro-inflammatory cytokines, upregulates Nrf2 and HO-1, and inhibits DOX-induced DNA damage in cardiac tissue [81]. Nerolidol (NERO) regulates the PI3K/Akt, Nrf2/Keap1/HO-1, and NLRP3/NF-κB/MAPK pathways for its cardioprotective effects [87]. Artesunate (ART) exhibits multi-organ protection by regulating NF-κB through multiple other pathways, with sufficient evidence from isoprenaline-induced cardiac hypertrophy rat models confirming that ART activates SIRT1 to inhibit NF-κB and ameliorate hypertrophy [28], while medium evidence from cerebral ischemia and I/R models shows direct NF-κB inhibition or TLR4/NF-κB blockade to reduce neuroinflammation [88,89]. Artemisinin and allicin combined decrease myocardium fibrosis in diabetic cardiomyopathy rats by inhibiting the NF-κB pathway, which has limited evidence from a single diabetic cardiomyopathy rat study for reducing myocardial fibrosis [13].

Diterpenoids also exhibit regulatory effects on the NF-κB pathway in the treatment of CVDs, with evidence strength ranging from sufficient multi-model validation to limited single-study data. Tanshinone IIA isolated from *Salvia miltiorrhiza* reduces AS plaque area, decreases serum inflammation levels, and inhibits the phosphorylation of ERK1/2, JNK, p38 and NF-κB p65 to improve atherosclerosis. It exhibits sufficient evidence across multiple models [91]. Rosmarinic acid is another compound in *Salvia miltiorrhiza*. Rosmarinic acid-functionalized micelles (PPS-PEG-RA@TA) have sufficient evidence from HFD-fed atherosclerotic mice for reducing plaque area and endothelial injury by downregulating VCAM-1 via NF-κB, with no observed toxic side effects and excellent biocompatibility [94]. Tanshinone IIA exhibits anti-inflammatory and neuroprotective effects in cerebral ischemia-reperfusion injury. It improves neurological function and reduces infarct volume by decreasing the expressions of L-1β, IL-6, TNF-α, TLR4 and MyD88, inhibiting NF-κB p65 phosphorylation and blocking IκB degradation [92,93]. Ovatodiolide from *Anisomeles indica* has a protective effect in ischemia-reperfusion-induced neuronal injury. It inhibits NF-κB, upregulates SIRT1 expression, and reduces brain edema, cerebral infarction area, neuronal apoptosis and microglial overactivation [99]. Neoandrographolide inhibits indexes of myocardial injury, infarct size, inflammatory cell infiltration, cell apoptosis, inflammatory cytokines, bax, cleaved caspase-3, P-IKBa, and P-NF-κB protein expressions and the translocation of NF-kB subunit p65 from the cytoplasm to the nucleus. These show a cardioprotective effect on I/R [96]. Carnosol and carnosic acid reduce cardiotoxicity (diesel exhaust particle-induced and LPS-induced, respectively) by inhibiting NF-κB and oxidative stress, with consistent results in mouse and poultry models but limited to a small number of independent studies [101,102]. Oridonin, the major diterpenoid from *Rabdosia rubescens*, prolongs cardiac allograft survival via NF-κB/NLRP3 inhibition, with medium evidence from mouse cardiac transplantation models showing reduced CD8^+^ T cell/macrophage infiltration and increased regulatory T cells (Tregs) [95]. Alterbrassicene A has limited evidence from a single study using human aortic valve interstitial cells and murine aortic valve stenosis models for inhibiting osteogenic calcification via NF-κB p65 phosphorylation suppression, representing a novel potential therapy for CAVD [100].

Natural triterpenoids are a class of terpenoid compounds with a basic skeleton composed of 30 carbon atoms, with evidence strength varying by compound from sufficient multi-model validation for myocardial protection and cardiac hypertrophy to limited single-study data for renal I/R injury and subarachnoid hemorrhage (SAH). Gypensapogenin I (Gyp I) is obtained from the hydrolysates of total gypenosides. It is investigated to have good myocardial protective activity. Strong evidence from in vitro and in vivo models backs this up. It increases the survival rate of H9c2 cells in vitro. It improves cardiac dysfunction, reduces collagen deposition and myocardial fibrosis (MF) in heart failure mice. Its mechanism is to regulate the TLR4/NF-κB/NLRP3 signaling pathway [109]. Cardiac hypertrophy increases the risk of myocardial ischemia, which finally leads to decreased myocardial contractility. Both lupeol and celastrol (CEL) can improve cardiac hypertrophy. Lupeol improves the morphological changes and cardiac dysfunction and remodeling in TAC mice and inhibits hypertrophic markers in neonatal rat cardiomyocytes, such as ANP, BNP, and β-MHC. The mechanism is to regulate the TLR4-PI3K-Akt signaling pathway to reduce inflammatory cytokines and NF-κB p65 nuclear translocation [104]. Celastrol (CEL) can improve cardiac hypertrophy. But it needs to be modified. ROS-responsive nanomicelles encapsulating celastrol realize an efficient and safe way to overcome the poor biocompatibility and low bioavailability of CEL. It is shown to regulate NF-κB signaling to inhibit pyroptosis and oxidative stress and then improve cardiac hypertrophy [105]. Betulinic acid (BA), a pentacyclic triterpene, has cardioprotective effects against chlorpyrifos-induced cardiotoxicity. It reduces cardiac function markers, tissue injury, pro-inflammatory cytokines and NF-κB [103]. Cycloastragenol (CAG) is the key active component of *Astragalus radix*. It has cerebroprotective effects on subarachnoid hemorrhage. These effects include inhibiting oxidative insults, inflammation, microglia activation, and neutrophil infiltration in the brain. The mechanism is to inhibit SIRT1 and inhibit NF-κB acetylation [107]. Cycloastragenol is an active ingredient of *Astragalus radix*. It reduces neuroinflammation in ischemic stroke by inhibiting NF-κB. It reduces M1 pro-inflammatory polarization and activates Nrf2/HO-1 to promote M2 anti-inflammatory polarization of microglia [108]. Celastrol can also regulate the Nrf2/HO-1 pathway. With regulation of the PI3K/AKT pathway, it inhibits NF-κB and ERK phosphorylation to reduce oxidative stress, inflammation, and apoptosis and finally exhibits a renoprotective potential against kidney ischemia-reperfusion injury, with limited evidence [106].

Tetraterpenes are terpenoids formed by linking eight isoprene units in either head-to-tail or non-head-to-tail arrangements. β-Cryptoxanthin is a common dietary carotenoid. It is reported to reduce myocardial ischemia-reperfusion injury in rats by inhibiting the expressions of p65 and p-p38 MAPK and reducing NF-κB-mediated inflammation. It shows medium evidence from a single major study with consistent histopathological and molecular results [112]. Astaxanthin has cardioprotective effects against isoprenaline-induced myocardial injury in rats, with limited evidence from a small number of experiments. It inhibits the TLR4/NF-κB signaling pathway to reduce oxidative stress and inflammation [113]. Lycopene exhibits protective effects against palmitate-induced cardiotoxicity besides of anti-lipidemic, antioxidant, cytoprotective, and anti-inflammatory effects. It decreases cardiac cholesterol, phospholipids, fatty acids, oxidative stress markers, and pro-inflammatory cytokines, while it increases triglycerides, antioxidants, and cardiac enzyme activities. It exhibits improvement of tissue damage and inflammatory cell infiltration in the heart [111]. Fucoxanthin reduces secondary brain injury after intracerebral hemorrhage in mice. It improves blood–brain barrier dysfunction and brain edema. The mechanism is to activate PI3K/Akt and inhibit the NF-κB signaling pathway. During the procedure, it reduces microglial M1 polarization and neuroinflammation [110]. Lycopene and fucoxanthin show limited evidence of tissue-specific NF-κB modulation.

Natural triterpenoid saponins are formed by a triterpene backbone with 30 carbon atoms and a sugar group. They usually include tetracyclic triterpenes and pentacyclic triterpenes. They are among the most extensively studied terpenoid subclasses for CVD treatment, with sufficient evidence for key compounds (astragaloside IV, asiaticoside) across multiple CVD models and medium-to-limited evidence for others (ginsenosides, ecliptasaponin A), all supported by in vitro and animal studies with no human clinical data. Asiaticoside (AT) is isolated from *Centella asiatica*. It can improve AS with sufficient evidence through reducing macrophage infiltration into plaques, including reducing atherosclerotic lesion formation in the aortic roots of ApoE-/- mice, and improves plaque stability. It is analyzed that it is related to RhoF proteasomal degradation and inhibition of NF-κB/MAPK signaling pathways [123]. Astragaloside IV exhibits sufficient evidence across a diverse range of CVD and ischemia/inflammation-related injuries. Its key mechanism is to regulate the NF-κB signaling pathway. Astragaloside IV improves cardiac dysfunction, hypertrophy, and fibrosis in high altitude hypoxia-induced cardiac injury. Meanwhile, it restores cardiac architecture and function with reducing oxidative stress and apoptosis. It mainly regulates two pathways, CaSR-NF-κB and EGFR-PI3K-AKT-MDM2 [115]. In acute myocardial infarction models, it targets MyD88 and inhibits the activation of the TLR4/MyD88/NF-κB pathway to reduce myocardial inflammation and fibrosis [114]. For pulmonary ischemia-reperfusion injury, astragaloside IV can specifically bind to the rhTLR4-MD-2 complex and inhibit inflammatory response mediated by the TLR4/MyD88/NF-κB p65 pathway to reduce injury [42]. Astragaloside IV can increase the survival rate of rats of sepsis-induced cardiac dysfunction due to accumulation of IKK-α and NF-κB p65 subunit phosphorylation [22]. Ginsenosides (20(R)-Rg3, Rb3, Rb1) exhibit neuroprotective effects in cerebral I/R injury and ICH via NF-κB inhibition, with medium evidence for each compound. Ginsenoside Rb1 can also regulate the TLR4/NF-κB pathway. It improves neurological deficits and learning-memory impairments in rats with intracerebral hemorrhage [116]. 20(R)-Ginsenoside Rg3 can inhibit the TLR4/MyD88/NF-κB signaling pathway to reduce neuroinflammation induced by cerebral ischemia/reperfusion injury [118]. Ginsenoside Rb3 also can regulate gut microbiota. It exhibits protective effects on cerebral ischemia/reperfusion injury by inhibiting neuroinflammation and ferroptosis through the NLRP3/NF-κB/GPX4 pathway [117]. Ciwujianoside C can activate NNAT, inhibit NF-κB, and block ferroptosis to protect against cerebral ischemia/reperfusion injury [119]. Ecliptasaponin A is the active component of *Eclipta prostrata* (Linn.). It has cardiac protective effects on acute myocardial infarction, reducing myocardial infarct size, decreasing myocardial cell apoptosis, inhibiting inflammatory cell infiltration, and improving cardiac function. It can directly bind to HMGB1 and inhibit the HMGB1/TLR4/NF-κB pathway to reduce inflammation [120]. Esculin and escin target NF-κB to reduce septic cardiomyopathy and cerebral ischemia-induced intestinal injury, respectively, with limited evidence from single rat studies. Esculin can bind to TLR4 and inhibit the phosphorylation of NF-κB p65. It can reduce septic cardiomyopathy with strong inflammation [48]. Escin decreases the cerebral ischemia-induced infarct volume, reduces the injury of the intestinal barrier, and decreases cort, endotoxin, and IL-1β levels through upregulating GR and inhibiting the NLRP3/MAPK/NF-κB pathway [122]. Platycodin D effectively reduces diabetic renal ischemia/reperfusion injury by AMPK/PINK1/Parkin-mediated mitophagy and inhibiting the MAPK/NF-κB pathway, with medium evidence from diabetic rat models and renal epithelial cells [121].

### 4.3. Phenylpropanoids

Phenylpropanoids are derived from phenylalanine. They include simple phenylpropanoids, coumarins, and lignans. A well-established body of preclinical evidence demonstrates that these compounds exert anti-inflammatory, antioxidant, and cardioprotective effects in cardiovascular disease (CVD) models, with the NF-κB signaling pathway as their core regulatory target. However, it is critical to note that nearly all supporting data to date derives from in vitro cellular assays and animal models, with no large-scale human clinical trials validating these effects.

Simple phenylpropanoids are characterized by a benzene ring linked to a three-carbon side chain. They exhibit NF-κB-inhibitory activities. Plantamajoside (PMS) is a major compound of *Plantago asiatica*. It increases cell viability in the myocardial I/R injury cell model. It can inhibit ROS production and pro-inflammatory cytokines release and increase superoxide dismutase (SOD), catalase (CAT) and glutathione peroxidase (GSH-Px) in multiple experimental replicates. The mechanism shows it to inhibit NF-κB activation and promote Akt/Nrf2/HO-1 antioxidant activation, with clear in vitro evidence for direct modulation of these two cascades [124]. Rosmarinic acid (RosA), a widely distributed polyphenol in Labiatae herbs, exhibits cardioprotective effects in murine in vivo myocardial I/R injury models (C57BL/6J mice) and in vitro HL-1 cardiomyocyte OGD/R models, with quantifiable reductions in infarct size, cardiac injury markers (CK-MB, cTnI), and ROS generation [125]. Its core mechanism is the suppression of NF-κB-mediated inflammatory signaling, including reduced phosphorylation of IκBα and NF-κB p65, with consistent results across both in vivo and in vitro systems [125]. In the pulmonary arterial hypertension (PAH) rat model, forsythoside B (FTS•B) improves monocrotaline-induced vascular remodeling, pulmonary artery pressure, right ventricular hypertrophy and survival. FTS•B reverses proliferation and migration of pulmonary artery smooth muscle cells and decreases PCNA and CyclinD1 expressions. It reduces IL-1β and IL-6 via inhibiting phosphorylation of p65, IκBα, IKKα and IKKβ [126]. Forsythoside B can also improve Kawasaki disease-related cardiac injury and HCAEC inflammatory injury via inhibiting pyroptosis through the SIRT1-NF-κB-p65 pathway, with in vitro evidence that the SIRT1 inhibitor EX 527 negates this effect [127].

Isofraxidin and fraxin exhibit neuroprotective effects in rat cerebral I/R injury models with consistent reduction in neuroinflammation, brain edema, and neurological deficits [129,130]. Coumarins are characterized by a benzo-α-pyrone skeleton. They can regulate the NF-κB signaling cascade. They exhibit anti-inflammatory and cytoprotective effects in different disease states. Isofraxidin has brain protective effects on cerebral ischemia reperfusion via inhibiting free radical and inflammation. It significantly downregulates COX-2, LOX-1, iNOS, TLR4 and NF-κB to reduce neuroinflammation and improve neurological deficit and brain edema in rats with ischemia/reperfusion (I/R)-mediated injuries [129]. Similarly, fraxin exhibits neuroprotective efficacy in cerebral ischemia/reperfusion injury too. It activates PPAR-γ, inhibits NF-κB, and enhances the Nrf2/HO-1 pathway of antioxidant defense [130]. Imperatorin shows anti-neuroinflammatory effects in ischemic stroke models by suppressing microglia activation and cytokine release through MAPK and NF-κB pathway inhibition, yet this promising mechanism rests solely on preclinical data with no human validation [41]. Esculin is a major active ingredient from *Cortex fraxini*. It is reported to protect cardiomyocytes against myocardial ischemia/reperfusion injury (MIRI). It inhibits NLRP3 inflammasome to reduce pyroptosis. It inhibits NF-κB phosphorylation and enhances Akt and GSK3β phosphorylation to block the GSK3β/NF-κB pathway [131]. Further analysis shows esculin can form stable hydrogen bonds by directly targeting and binding to TLR4 and inhibits downstream NF-κB activation to reduce sepsis-induced myocardial inflammation, oxidative stress and apoptosis [48]. However, all evidence remains confined to rat models and cell cultures, with no human clinical data to confirm whether these molecular interactions translate into tangible cardiac benefits for patients.

Lignans are characterized by a unique chemical structure where two phenylpropanoid units (C6-C3) are connected at their β-carbons (C8-C8′). A variety of natural lignans exhibit significant protective effects in cardiovascular and neuroinflammatory diseases by NF-κB. Phillyrin is a well-known natural compound from the dried fruits of *Forsythia suspensa* (Thunb.) Vahl. It can improve cardiac function and reduce inflammatory response, cardiac hypertrophy and hypertrophic markers. It inhibits the phosphorylation of p38 MAPK, ERK1/2, AKT, and NF-κB p65 in heart tissue while reducing ROS production [46,132]. Only phillyrin has early safety data from preclinical work showing it is well-tolerated [132]. However, phillyrin shows low bioavailability with only mild and transient adverse effects [132]. Syringaresinol (SYR) exhibits cardioprotection in MI models with differing evidence comprehensiveness. SYR alleviates MI-induced cardiac dysfunction, infarct size, hypertrophy, fibrosis, inflammation, and apoptosis in in vivo and in vitro models, with network pharmacology and experimental validation confirming reversal of key protein expression (AKT1, EGFR, CASP3, NFKB1, etc.) [135]. Schisantherin A has protective effects on cardiac injury in the ISO-induced MI model of rats. During the procedure, it activates the PI3K-AKT/Nrf2/ARE pathway and inhibits the TLR4/MAPK/NF-κB pathway. Schisantherin A shows good inhibitory effects on oxidative stress and inflammation [137]. Pinoresinol diglucoside (PDG) is the active compound isolated from *Eucommia ulmoides*, *Styrax* sp. and *Forsythia suspensa*. PDG can reduce cardiac histomorphology damages, fibrosis, and inflammation and decrease upregulations of hypertrophic biomarkers via inhibiting AKT/mTOR/NF-κB activation. It prevents cardiac histomorphological damage, fibrosis, and inflammation by inhibiting AKT/mTOR/NF-κB activation, with dose-dependent efficacy in both in vivo and in vitro systems [138]. Tetrahydrofurofuranoid lignans are isolated from *Magnoliae flos*. They have limited but promising preclinical evidence for broad cardioprotective effects. They can inhibit NF-κB, MAPK and other pathways. It has cardioprotective effects through inhibiting the cAMP/PKA pathway and treating atherosclerosis by downregulating the TLR4/NF-κB signaling pathway [133]. It is important to know that not all lignan compounds have cardiovascular protective effects. Podophyllotoxin’s primary activity is antitumor, rather than cardiovascular protection. It induces cardiotoxicity with cardiac energy metabolism, elevation of myocardial enzymes and pathological alterations in rat hearts via the SIRT1/PPAR/NF-κB axis [136]. This highlights the need for rigorous structural and functional characterization of lignans, as structural similarity does not equate to shared cardioprotective effects.

### 4.4. Alkaloids

Alkaloids are a class of nitrogen-containing organic compounds. They exhibit alkaline properties. They are nitrogen-containing organic compounds excluding amino acids, proteins, peptides, nucleic acids, nucleotides, and amino sugars in biological organisms. Most of them are distributed in dicotyledons, such as Ranunculaceae, Papaveraceae, Menispermaceae, Solanaceae, and Berberidaceae.

Berberine exerts cardioprotective effects in sepsis-induced myocardial injury and calcified aortic valve disease (CAVD), with multiple animal and in vitro studies confirming its efficacy. It inhibits TLR4, p65, TNF-α, and IL-1β and reduces cTnT and myocardial cell swelling in sepsis-induced myocardial injury in rats [19]. Berberine can inhibit the Smad1/5/8 and NF-κB pathways to reduce inflammation, osteogenic differentiation alkaline phosphatase activity and calcified nodule formation of aortic valve interstitial cells under osteogenic conditions, meaning it is of benefit in calcified aortic valve disease [139].

Tetrandrine, a bisbenzylisoquinoline alkaloid, shows protective effects on ventricular remodeling and atherosclerosis, with robust evidence from multiple rodent models and in vitro cellular studies. It has anti-ventricular remodeling effects to reduce hypertensive heart failure. It reduces Ang II- or TAC-induced myocardial hypertrophy, fibrosis and inflammation by inhibiting the MAPK/NF-κB pathway [4]. Leonurine’s effects are similar to that of tetrandrine [156]. Tetrandrine reduces vitamin D3/high cholesterol diet-induced atherosclerosis in rats. It reduces the serum lipids and inhibits inflammation and oxidative stress. The mechanism is to regulate the miR-34a and Wnt5a/Ror2/ABCA1/NF-κB pathway [140]. Its efficacy in this context is comparable to atorvastatin in rodent models.

Corynoline improves Ang II-induced hypertensive heart failure without altering blood pressure. It inhibits inflammation, myocardial fibrosis, and hypertrophy to prevent heart dysfunction. It increases PPARα and enhances the interaction between PPARα and p65 to inhibit the NF-κB pathway [154]. Similar to lycorine, it improves hypertensive heart failure by reducing angiotensin II-induced myocardial hypertrophy, fibrosis and inflammation. NF-κB, PI3K, and AKT are its key target proteins to inhibit inflammation and protect the heart in mice [145]. Anatabine prevents cardiac structural remodeling, reduces sympathetic activation and inflammation, and decreases blood pressure to protect cardiovascular activity in hypertensive rats. Its mechanism is to decrease NF-κB activity, NLRP3-dependent inflammasome, pyroptosis, and ROS production in the hypothalamic paraventricular nucleus of hypertensive rats [149].

Oxymatrine reduces isoproterenol-induced inflammation and heart failure injury, with evidence restricted to in vitro and animal studies. It reduces ISO-induced cardiac injury, myocardial necrosis, interstitial edema, and fibrosis. It inhibits the TLR4/NF-κB and MAPK pathways to decrease TNF-α and IL-6 levels [153]. Matrine exhibits anti-inflammatory and anti-remodeling effects. It inhibits hypoxia-induced abnormal proliferation of pulmonary artery smooth muscle cells and pulmonary vascular remodeling. It is related to regulating the RPS5/NF-κB pathway [148]. Matrine combined with tacrolimus reduces acute rejection in murine heart transplantation in mice. They inhibit dendritic cell maturation and inflammatory responses mainly by the ROS/ERK/NF-κB pathway [144].

Rutaecarpine is an indolopyridoquinazolinone alkaloid isolated from *Evodia rutaecarpa*. It has anti-inflammatory and anti-thrombotic effects in cardiovascular-related models, with evidence from in vitro macrophage studies and mouse thrombosis models. It exhibits anti-inflammatory effects in LTA-stimulated RAW macrophage cells. The mechanism is to reduce the release of inflammatory mediators by inhibiting the NF-κB and ERK/p38 signaling pathways [151]. Rutaecarpine decreases mortality in adenosine diphosphate (ADP)-induced acute pulmonary thromboembolism mice. It does not affect the bleeding time. During the procedure, it regulates p38 to inhibit IKK and p65 phosphorylation, reverse IκBα degradation, and finally inhibit p65 activation [152]. Piperlongumine can reduce the phosphorylation levels of key molecules in the NF-κB signal pathway to improve cardiomyocyte injury and enhance cardiac function. Its efficacy is supported by a single VMC mouse model and in vitro HL-1 cell study [146].

Colchicine exhibits significant protective effects in many cardiovascular diseases, with sufficient and multifaceted cardioprotective evidence. The mechanisms are related to regulating the NF-κB signaling pathway. In the myocardial injury mice model, colchicine reduces myocardial injury, fibrosis, neutrophil infiltration, and cardiomyocyte pyroptosis to improve cardiac function by regulating the ESR1-PI3K-Akt-NF-κB pathway [43]. In the atherosclerosis combined with diabetes mellitus mice model, colchicine reduces the atherosclerotic plaques area in the aorta and aortic root. It decreases intraplaque macrophage accumulation with enhancing plaque stability and reduces inflammation PIM2 and the PP65/P65 ratio [23]. Colchicine phosphatidylserine-exposing nanovesicles (Col@PSVs) enlarge the narrow therapeutic window by reducing dose-dependent toxicity and enhance anti-inflammatory efficacy. This inhibits CCR7-mediated NF-κB signaling activation in foam cells to improve plaque stability and decrease inflammatory cytokine release [141]. In the ApoE-/- atherosclerotic mouse model, a novel delivery nanosystem encapsulated with colchicine (VHPK-PLGA@COL) significantly reduces the atherosclerotic plaque burden and improves plaque stability by blocking NF-κB/NLRP3 inflammatory pathways [142]. These nanosystems are still in preclinical stages with no human trials.

Tetrahydropalmatine (THP) is the main component of *Corydalis yanhusuo*. It induces the polarization of M1 macrophage to M2 and inhibits inflammation to reduce limb ischemia-reperfusion-induced acute lung injury. The pathway is related to TLR4/NF-κB/NLRP3. Its effect is supported by limited evidence from a single rat model and an in vitro coculture study, with no investigation into direct cardiac protection [147]. Koenigicine provides preventive, suppressive, and ameliorative effects at all stages of myocardial infarction through anti-inflammatory and antioxidant effects. It regulates NF-κB/HO-1/NQO-1 pathways. This efficacy, backed by limited evidence from a single isoproterenol-induced MI rat model, lacks replication or mechanistic validation in other systems [155]. SGK1 is one of the regulators of NFκB and is involved in the fibrotic effects of angiotensin II and aldosterone, and cardiac fibroblasts differentiation. Boldine inhibits its activation to significantly reduce inflammation [148]. Gramine, a natural indole alkaloid, can be found in many plants. It can improve sepsis-mediated myocardial dysfunction in mice. The mechanisms show that it inhibits NF-κB p105 ubiquitination to block NF-κB p105 changed into p50. This mechanism is supported by moderate evidence from mouse sepsis models and in vitro primary cardiomyocyte studies, which demonstrate high target-binding accuracy but have not been validated in human sepsis [30]. Anisodamine (654-1/654-2) reduces myocardial injury in septic shock rats. 654-1 blocks the NF-κB/NLRP-3 pathway, and 654-2 activates PI3K-AKT by inhibiting NF-κB, together reducing inflammation and apoptosis [150]. No human septic shock clinical data exist for either anisodamine formulation.

Capsaicin exerts protective effects in hypertension, cardiac hypertrophy, and aortic valve interstitial cell (VIC) calcification, with evidence from spontaneous hypertensive rat (SHR) models and in vitro VIC studies (evidence level: moderate) and no human clinical trials. In SHRs, it inhibits oxidative stress and inflammation in the hypothalamic paraventricular nucleus (PVN) by increasing SIRT1 and GAD67 levels, decreasing ROS production, p-IKKβ, pro-inflammatory cytokines, and MAPKs pathway protein expression [44]. Capsaicin reduces aortic valve interstitial cells (VICs) calcification induced by pro-calcifying medium (PCM). This is because it reduces calcium deposition level, downregulates calcification markers, including Runx2, osteopontin, and BMP2. It also inhibits the redox-sensitive NFκB/AKT/ERK1/2 signaling pathway. Its effects on calcific aortic valve stenosis (CAVS) are limited to in vitro studies [50]. Vincristine (VCR) is a vinca alkaloid. It significantly reverses ISO-induced cardiac hypertrophic phenotypes. The mechanism is related to blocking the ROS/NO/NF-κB signaling pathway to inhibit oxidative inflammation and apoptosis. This cardioprotective effect rests on limited evidence from a single rat model, and its clinical application remains confined to oncology without any cardiovascular translational follow-up [47].

### 4.5. Quinones

Quinones are characterized by a cyclic diketone structure, including benzoquinones, naphthoquinones, phenanthraquinones, and anthraquinones. They exhibit positive effects on the treatment of cardiovascular and neurological diseases via the NF-κB pathway, with evidence for these effects currently derived primarily from in vitro cellular models and in vivo animal experiments.

Emodin, anthraquinone, and physcion are the main chemical constituents of rhubarb, belonging to anthraquinone, and emodin has the most sufficient evidence for its anti-atherosclerotic effect among quinone compounds, supported by multiple in vitro and in vivo experimental studies. Emodin reduces atherosclerotic lesions in HFD-treated ApoE-/- mice, and this effect is consistently verified in PMA-induced human mononuclear cell line THP-1 macrophages in vitro. It decreases the expression of NLRP3, GSDMD, IL-1β, and IL-18 in vitro and in vivo. In addition, it significantly blocks NF-κB activation by inhibiting TLR4/MyD88 complex formation [161]. Aloe-emodin, another anthraquinone component of rhubarb, exerts neuroprotective effects in neurological disease models, and its protective mechanisms for neurons have been confirmed by multiple independent vivo and in vitro studies with consistent results. It inhibits microglia activation and promotes transformation from M1 phenotype to M2 at the stage of early brain injury after subarachnoid hemorrhage. Its mechanism is related to the NF-κB and PKA/CREB pathways to reduce inflammatory response and neuron injury [162]. It is consistent with the previous results that aloe-emodin has protective effects on neurons. It reduces nerve injury, oxidative stress and neuroinflammation by activating PI3K/AKT/mTOR and inhibiting the NF-κB pathway in the middle cerebral occlusion reperfusion rats in vivo and oxygen and glucose deprivation reperfusion, and in LPS-stimulated cells in vitro [163]. Physcion exerts neuroprotection against cerebral I/R injury, and this effect is supported by in vitro OGD/R cell models and in vivo rat cerebral I/R models, but the relevant research is limited to a single study with moderate evidence intensity. It reduces oxidative stress and pro-inflammatory cytokine production by inhibiting TLR4/NF-κB [164].

Thymoquinone exerts neuroprotective and cardioprotective effects by regulating the NF-κB pathway, and its protective effects against drug-induced and chemical-induced neurotoxicity and cardiotoxicity are confirmed by multiple in vivo rat model studies, with moderate to sufficient evidence intensity. It reduces neurotoxicity, neuroinflammation and striatal dysfunction in hypertensive dams and their F1 male offspring induced by a bisphenol A analog mixture by regulating the p53/NF-κB apoptotic pathway [157]. It also reduces prilocaine-induced neurotoxicity, epileptiform activity and cardiotoxicity by inhibiting NF-κB activation, reducing oxidative stress and AQP4 expression [158].

Naphthoquinone exhibits neuroprotective effects in cerebral ischemia/reperfusion injury models, and the relevant evidence is derived from a single study that simultaneously used in vitro OGD/R BV2 microglial cellular models and in vivo middle cerebral artery occlusion/reperfusion rat models, with moderate evidence intensity for its anti-cerebral I/R injury effect. It inhibits neuroinflammation, microglial polarization, and NF-κB signaling activation by regulating the NOD2/RIP2/NF-κB pathway to reduce cerebral ischemia/reperfusion injury [159].

### 4.6. Steroids

Steroids include plant sterols, bile acids, C21 steroids, insect molting hormones, cardiac glycosides, steroidal saponins, steroidal alkaloids, bufadienolides, etc.

Dioscin is a kind of steroidal saponin and exhibits protective effects on sepsis-induced cardiomyopathy (SIC), with evidence limited to a single study that combined in vivo rat SIC models and in vitro H9c2 cell experiments, and no human study or multiple independent animal validations are available. It decreased the heart rate and heart weight index, increased left ventricle ejection fraction and mean arterial blood pressure, and reduced the myocardial tissue damage and 4-hydroxy-2-nonenal level in SIC rats. The mechanism shows it is related to inhibiting the TLR4/MyD88/p65 pathway to reduce ROS production and enhance antioxidant [165]. The TLR4/MyD88/p65 pathway as its core mechanism is verified in both cellular and animal models of SIC, with consistent in vitro and in vivo mechanistic evidence, yet lacking cross-species validation.

Saponins from *Allium macrostemon* bulbs (SAMB) belong to steroidal saponins. They exhibit beneficial effects on endothelial inflammation, acute lung injury and atherosclerosis, with multiple independent in vivo and in vitro studies supporting its efficacy for these conditions, representing moderate to sufficient evidence for cardiovascular and inflammatory disease-related indications. It inhibits the NF-κB pathway to reduce VCAM-1 expression and monocyte adhesion [166]. They can inhibit inflammation and atherosclerosis plaque formation in apolipoprotein E deficiency (ApoE-/-) mice with high-fat diet feeding. They inhibit oxidized-LDL-induced foam cell formation by downregulating CD36 expression and then inhibit lipid endocytosis in bone marrow-derived macrophages. This is related to regulating the NF-κB/NLRP3 pathway. Its anti-atherosclerotic effect is well-supported by in vivo murine atherosclerosis models and in vitro macrophage experiments, with both phenotypic and mechanistic validation providing sufficient preclinical evidence for atherosclerosis [167]. Inflammation is a major risk factor for cardiovascular disease including atherosclerosis. Steroid compounds from *Solidago canadensis* show broad-spectrum anti-inflammatory effects, with evidence from in vitro cellular models (RAW264.7 cells, BMDMs, PBMCs) and in vivo murine inflammatory/colitis models representing moderate preclinical evidence for inflammatory cardiovascular diseases such as atherosclerosis, and no human study data is available. They activate AMPK and simultaneously inhibit NF-κB/NLRP3 pathways and are hoped to be used in various inflammatory diseases [168].

### 4.7. Polyphenols

Here, the protective effects of phenolic natural products on CVDs are discussed, with a critical analysis of the evidence strength for distinct CVD indications and a clear distinction between in vitro studies, animal experiments, and the currently limited human research evidence for all compounds reviewed. Common phenolic natural products include resveratrol, curcumin and salvianolic acid, etc., with their cardioprotective effects primarily validated in preclinical models and varying levels of evidence support for different CVD subtypes.

Resveratrol and its analogs can prevent and treat liver injury, vascular diseases, hypertension, obesity-related cardiovascular complications and diabetic cardiomyopathy by multi-pathway regulation with NF-κB, with moderate to strong preclinical evidence from in vivo and in vitro studies. Resveratrol reduces thioacetamide-induced liver fibrosis, inflammation and systemic hypertension by inhibiting TNF-α/NF-κB/iNOS/HIF-1α [169]. Both in vitro human cell experiments and in vivo mouse models demonstrate that resveratrol can reduce vascular endothelial inflammation, monocyte adhesion and early atherosclerotic lesions by inhibiting NF-κB activation [170]. Rat model studies alone confirm that resveratrol can reduce cyclosporin A-induced vasoconstriction and drug-induced hypertension. The mechanism is to activate AMPK/SIRT1 and inhibit the MAPK/NF-κB pathway [171]. It has improvement effects on HFD-induced obesity and associated vascular endothelial dysfunction (VED) and myocardial infarction (MI) in Wistar rats. It significantly lowers cardio and vascular damage and inhibits the TLR4/MyD88/NF-κB/iNOS pathway [172]. Its derivative polydatin inhibits the hyperglycemia-induced myocardial injury and inflammatory fibrosis of diabetic cardiomyopathy models in vivo and in vitro. It upregulates Caveolin 1 to inhibit the NF-κB pathway [182], though evidence for polydatin remains limited to a small number of preclinical studies to date.

Multiple rat model studies confirm that curcumin exerts protective effects against doxorubicin-induced cardiotoxicity via multiple NF-κB-related mechanisms. It downregulates iNOS, NF-κB, and TNF-α [173], upregulates Apelin/NF-κB [174], and blocks Rac1/TWEAK/Fn14/NF-κB, with consistent results across these studies demonstrating robust inhibition of oxidative stress, inflammation, and apoptotic signaling in cardiac tissues [175]. The curcumin analog JM-2 provides strong protection against diabetic cardiomyopathy. It prevents cardiac functional and structural deficits effectively and reduces cardiac inflammation and fibrosis. It inhibits NF-κB to reduce inflammation [49]. This study represents the only dedicated investigation of JM-2 to date, with highly consistent in vivo and in vitro evidence supporting its potential for DCM.

Other phenolic natural products also prevent and treat atherosclerosis, drug-induced cardiotoxicity, pulmonary hypertension, acute myocardial infarction and pancreatic ischemia-reperfusion injury by regulating NF-κB or upstream/downstream pathways, with evidence strength ranging from limited single-study data to moderate consistent preclinical evidence across different compounds and CVD indications.

A single combined mouse model and human cell study confirms that gastrodin inhibits atherosclerosis progression by regulating lipid profiles and inhibiting inflammation. It is related to inhibition of the TLR4/NF-κB pathway, providing limited but consistent preclinical evidence for atherosclerotic disease [181]. Multiple in vitro PAEC experiments and in vivo rat model studies confirm that salidroside reduces hypoxia-induced pulmonary artery endothelial cell apoptosis and pulmonary hypertension. It inhibits the AhR/NF-κB inflammatory pathway and activates the Nrf2/HO-1 antioxidant pathway [176]. A single in vitro H9C2 cell study shows that salvianolic acid A protects cardiomyocytes against doxorubicin-induced cardiotoxicity. It inhibits the NF-κB pathway activation to decrease lncRNA PVT1 level and reduce cardiomyocytes injury and apoptosis, with limited evidence for drug-induced cardiotoxicity limited to this in vitro investigation [180]. Velutin is good for treating abamectin induced cardiotoxicity in rats. It simultaneously inhibits the JAK1/STAT3 and NF-κB pathways and activates the Nrf2/Keap1 antioxidant pathway to reduce cardiac oxidative, inflammatory and apoptotic injuries induced by abamectin [177]. Total xanthones from Gentianella acuta, which is an ethnomedicine with distinctive ethnic characteristics, reduce acute myocardial infarction, cardiomyocyte pyroptosis and inflammation by targeting BRD4 to inhibit the TLR4/NF-κB/NLRP3 pathway, with evidence limited to a single comprehensive preclinical study combining in vivo and in vitro experiments, providing novel but unvalidated evidence for acute myocardial infarction [179]. Oleuropein (OLP) is a naturally phenolic compound in olive plants. A single rat model study shows that it can reduce pancreatic injury persuaded by ischemia-reperfusion (I/R). It reduces oxidative stress and inflammation by inhibiting the HMGB1/NF-κB pathway, with limited preclinical evidence for ischemia-reperfusion injury in pancreatic tissue, with extrapolation to cardiac I/R injury remaining uninvestigated [178].

### 4.8. Saccharides

Carbohydrates, ranging from monosaccharides to complex polysaccharides, are benefit to cardiovascular protection targeting the NF-κB signaling pathway, with varying levels of preclinical evidence supporting their efficacy across distinct cardiovascular pathologies and human clinical research for this class of compounds remaining currently limited.

The monosaccharide *L*-fucose has significant cardioprotective effects, with evidence from a single in vivo mouse model and complementary in vitro AC16 cell experiments fully demonstrating its ability to improve cardiac function by inhibiting inflammation, pyroptosis and mitochondrial injury in obese hearts. The mechanism is to inhibit the TLR4/MyD88/NF-κB pathway [183].

Polysaccharides isolated from traditional Chinese medicinal herbs also exhibit benefits for various cardiovascular pathologies, with most supporting evidence derived from animal models and in vitro cellular assays, and no human study data available to date. For example, astragalus polysaccharides (APSs) exhibit protective effects against transport stress-induced cardiac injury by blocking the mtDNA-PRRs/NF-κB/NLRP3/cGAS-STING pathway. This finding was validated by a single in vivo chick model, which carries limited translational relevance to mammalian systems [185]. ASPs reduce myocardial injury of DCM rats by activating the AMPK/PPAR-γ signaling pathway and inhibiting the NF-κB pathway. This cardioprotective effect has been confirmed in a rodent DCM model, providing moderate preclinical evidence for diabetic cardiomyopathy intervention [184].

Poria cocos polysaccharides (PCPs) are one of the main active ingredients of Poria. They can reduce atherosclerosis by reducing blood lipids and vascular inflammation. This is related to inhibition of the TLR4/NF-κB pathway, with this anti-atherosclerotic effect supported by in vivo ApoE-/- mouse model experiments, representing moderate preclinical evidence for atherosclerotic disease [187]. It is similar to the polysaccharide component (PCP1) from *P. cyrtonema* Hua. It shows anti-atherosclerotic effects, with multiple in vitro macrophage assays and in vivo ApoE-/- mouse studies providing sufficient preclinical evidence for its dual regulatory effects on lipid metabolism and inflammation. It inhibits CD36/MSR1-mediated lipid uptake and blocks TLR4/NF-κB [186].

Citri Reticulatae Pericarpium polysaccharide CP-0 is a novel triple-helical heteropolysaccharides containing glucose, ribose and mannose. It reduces LCWE-induced endothelial dysfunction by targeting TLR2 to block NF-κB-NLRP3 inflammasome activation [188]. This protective effect on endothelial function was validated by in vitro mouse microvascular endothelial cell experiments, constituting limited preclinical evidence with no in vivo animal model confirmation for coronary arteritis or endothelial dysfunction.

*Ganoderma lucidum* polysaccharides and triterpenoids reduce endothelial oxidative stress and macrophage inflammatory polarization delay atherosclerosis by inhibiting the NF-κB/LOX-1 and Notch1 pathways [189]. This effect is supported by robust preclinical evidence from both in vivo high-fat diet rabbit models and in vitro human umbilical vein endothelial cell/macrophage assays, making it one of the best-characterized traditional Chinese medicinal polysaccharide combinations for atherosclerotic intervention to date.

### 4.9. Tannins

Tannins are usually found in various medicinal plants and fruits, and multiple animal experimental studies have sufficiently confirmed their cardioprotective effects through inhibition of the NF-κB signaling pathway.

Punicalagin is the major ellagitannin isolated from pomegranates. It exhibits significant protective effects against isoproterenol-induced myocardial infarction in rats. It reduces oxidative damage, inflammation, and apoptosis. It effectively reduces myocardial injury markers, including CK-MB and cardiac troponin I, while it inhibits pro-inflammatory cytokines, including TNF-α, IL-6, and IL-1β. It inhibits the NF-κB pathway and activates the Nrf2/HO-1 antioxidant defense system [190]. This is similar to the previous article report that tannins from *Punica granatum* L. inhibit NO production due to key groups like HHDP, flavogallonyl and/or gallagyl. They simultaneously inhibit the p38 MAPK and NF-κB pathways to decrease the levels of inflammation-related cytokines and mediators, such as IL-6, TNF-α, iNOS and COX-2 [191]. This anti-inflammatory mechanism of pomegranate tannins is supported by both in vivo animal and in vitro cell experiments, with moderate overall evidence strength.

Corilagin, another natural tannin, has been robustly demonstrated to exert anti-atherosclerotic effects in HFD-induced rabbit atherosclerosis models. It significantly reduces the serum levels of TC, TG and LDL-C, increases the HDL-C levels, decreases the intimal thickening in the thoracic aorta, and reduces the formation of foam cells in an HFD-induced rabbit atherosclerosis model. It is verified to have anti-atherosclerotic effects by inhibiting the LOX-1/MyD88/NF-κB pathway [192]. The evidence for corilagin’s protection against chemotherapeutic drug-induced cardiotoxicity remains limited (only one in vivo animal study reported), and further in vivo replication and in vitro mechanistic studies are required to validate this effect.

### 4.10. Other Compounds

Besides the above categories, some of other classes of compounds are also of benefit to prevent and treat CVDs by regulation of the NF-κB pathway. Carvacrol exhibits cardioprotective effects in septic-myocardial injury mice, including improving cardiac function, reducing inflammatory cytokines, decreasing oxidative stress, and inhibiting cardiomyocyte apoptosis. Multiple in vitro and in vivo studies have confirmed that carvacrol significantly inhibits the expression of TLR4, MyD88, and NF-κB, supporting a moderate level of evidence for its role in ameliorating sepsis-induced myocardial dysfunction [193]. Lauric acid (LA) is a medium-chain saturated fatty acid presented in coconut oil. It can improve doxorubicin-induced cardiac injury in rats. It reduces oxidative stress and inflammation, improves biochemical values, preserves myocardial structure and restores the antioxidant status in the DOX-induced cardiotoxicity model, with a single animal study demonstrating that LA inhibits NF-κB p65 to reduce pro-inflammatory cytokine levels, representing limited evidence for its cardioprotective effect against chemotherapeutic-induced cardiac damage [194].

All-*trans*-retinoic acid has neuroprotective effects by reducing neuroinflammation after cerebral ischemia-reperfusion. It minimizes cerebral infarct volume and reduces neurological deficit scores, cerebral edema, and pro-inflammatory cytokines after stroke. Multiple in vivo rat model studies have verified that its neuroprotective mechanism is linked to blocking microglial M1 polarization and activating the M2 phenotype via inhibition of the TLR4/NF-κB signaling pathway, providing a moderate level of evidence for its efficacy in ischemic stroke-related neuroinflammation, a key complication of cardiovascular ischemic events [195].

### 4.11. Traditional Chinese Medicine Formula and Prescription

Traditional Chinese medicine (TCM) formulas and natural product combinations have the advantages of preventing and treating CVDs through multi-component and multi-pathway regulatory effects centered on the NF-κB signaling pathway, with most evidence currently derived from in vitro cell experiments and in vivo animal models and limited human clinical research data available for these formulations to date.

For example, the baicalin and geniposide combination (BC/GD) is good for improving chronic cerebral ischemia (CCI) and kidney injury, with moderate evidence strength supported by both in vitro and in vivo rat experiments. BC/GD improves cognitive impairment, increases cerebral blood flow, and inhibits microglia activation and polarization of pro-inflammatory phenotypes to reduce inflammation in rats. In vitro and in vivo experiments show that it increases the expression of HIF-1α and EPOR and reduces the phosphorylation of NF-κB [196]. Another example, beta-caryophyllene combined with L-arginine, can improve streptozotocin-induced diabetic cardiomyopathy in rats, with evidence limited to a single in vivo rat study. This combination recovers the hemodynamic parameters and low glucose level, cardiac markers, IL-6, and TNF-α levels. It is suggested to inhibit the NF-κB pathway, with no in vitro or follow-up validation studies reported [197].

In addition, Guyuan Jiannao Decoction (GYJND) is a traditional Chinese formula containing lavonoids, diterpenoids, triterpenoids, saponins, and phenolic acids. It is usually used for treating cerebral small vessel disease (CSVD) in clinics. In the CSVD rat model, GYJND improves cognitive function, the permeability of the blood–brain-barrier (BBB) and microvascular structure, and reduces brain tissue injury, the morphology and structural damage of the neurovascular unit (NVU). GYJND also inhibits neuronal apoptosis. It increases the NeuN, GFAP and decreases the Iba-1, AQP4 level in the prefrontal cortex and hippocampus by activating PI3K/AKT and inhibiting the NF-κB pathway [198]. Similarly, Taohong Siwu decoction improves AS in rats by inhibiting AS progress and inflammatory cytokines in plasma. This may be related to inhibiting TLR4, MyD88 and NF-κB p65 levels. This mechanism is supported by moderate evidence combining in vivo protein expression assays with in silico molecular docking predictions [199].

Gegen Qinlian Pills (GQP) is included in the Chinese Pharmacopoeia. GQP reduces inflammation-related thrombosis, containing increasing tail blood flow, decreasing thrombi in the lung, liver, and tail, due to it reducing the adhesion of platelets and inhibiting the HMGB1/NF-κB/NLRP3 signaling pathway [200]. These mechanisms are validated by moderate evidence from in vivo mouse models and in vitro human endothelial cell studies, with clinical relevance for hyperinflammatory conditions.

Xueshuantong (XST) injection is composed of saponins from *Panax notoginseng*. It significantly improves the neurological damage, inflammatory infiltration, and microvessel morphology. Also, it increases microvessel density in the brain of middle cerebral artery occlusion/reperfusion (MCAO/R) rats. The mechanism is related to its inhibition of JAK2, STAT3, IκB, NF-κB and JNK phosphorylation to reduce the expression of inflammatory mediators, with its signaling pathway regulation fully verified via both in vivo and in vitro phosphorylation assays and agonist intervention experiments [201].

## 5. Safety Evaluation and Clinical Evidence of Natural Products in CVDs

### 5.1. Safety Evaluation

Natural products usually exhibit safety in treatment of CVDs. For example, curcumin, as the active component from turmeric, exhibits safety with minimal adverse effects [2]. Artesunate shows dose-dependent cardioprotective effects during treating cardiac hypertrophy [28]. Berberine has been confirmed safe in animal models at therapeutic doses during treating septic cardiomyopathy [19]. But some compounds require attention. Colchicine’s clinical application is limited by a narrow therapeutic window, while the change of delivery systems reduces dose-dependent toxicity [141]. Generally, most natural products such as astragaloside IV [22], luteolin [7], and quercetin [21] exhibit good biocompatibility in preclinical studies, and no severe organ damage are reported for them.

### 5.2. Current Clinical Research Progress

Increasing clinical evidence of natural products treating CVDs has been shown in recent years. For example, resveratrol, abundant in red wine, shows vascular benefits in human trials, although its high dosage exhibits fewer effects than food-sourced supplementation [15]. Sodium tanshinone IIA Sulfonate belongs to clinical cardiovascular medicine. It can reduce diabetic vascular senescence via regulating inflammatory responses [6]. Wogonoside and tanshinone IIA ameliorate atherosclerosis via inhibiting the TLR4/NF-κB pathway [35,91].

While most research remains in preclinical stages, hesperidin [12] and ginsenoside Rb1 [116], for example, exhibit positive effects on pulmonary arterial hypertension and intracerebral hemorrhage in animal models. Further clinical studies are needed to validate their efficacy for preventing and treating cardiovascular diseases.

## 6. Structure–Activity Relationship, Delivery System, and Combination Therapeutic Strategies

### 6.1. Structure–Activity Relationship

Natural compounds with different structures affect the efficacy of treating CVDs via specific molecular interactions. Kaempferol-3-O-rutinoside is a flavonoid glycoside from *Carthamus tinctorius*. It exhibits cardioprotective activity and the rutinoside moiety binds NLRP3, caspase-1, and GSDMD to inhibit NF-κB/NLRP3/caspase-1 pathway activation and ameliorates ventricular remodeling post-myocardial infarction [70]. Curcumin analog JM-2 is structurally changed from curcumin, which belongs to the parent polyphenol. It enhances NF-κB inhibitory effects in diabetic cardiomyopathy and provides stronger protection against cardiac inflammation [49]. Sodium tanshinone IIA sulfonate (STS), a water-soluble derivative of the lipophilic tanshinone IIA, regulates the A20-NFκB-NLRP3 inflammasome pathway and reduces diabetic vascular senescence effectively [6].

### 6.2. Delivery System Optimization Strategies

Nanotechnology delivery systems have emerged as a pivotal approach to overcoming the poor bioavailability of natural NF-κB inhibitors, with a series of engineered nanoplatforms and modified extracellular vesicles demonstrating promising preclinical performance and laying essential foundations for clinical translation, albeit with distinct limitations remaining to be addressed for practical clinical application [25,51,97,105,142].

KPF@MM-NPs, a macrophage-biomimetic kaempferol delivery platform, achieves effective targeting of atherosclerotic lesions with favorable biosafety; this nanosystem has completed in vivo validation in ApoE-/- mice and its anti-atherosclerotic (anti-AS) mechanism has been clearly elucidated, yet its large-scale preparation process and long-term in vivo retention characteristics require further optimization [51]. VHPK-PLGA@COL, a colchicine-loaded nanoparticle system, enhances biosafety and enables sustained drug release, while its surface modification with VHPK peptide endows specific targeting of inflammatory endothelial cells, thereby ameliorating atherosclerotic plaque accumulation and reducing the adverse effects of colchicine [142]; it has exhibited enhanced anti-AS efficacy and reduced safety risks in mouse models, effectively addressing the clinical translational bottlenecks of colchicine, though the stability of its peptide modification and drug release rate need tailored adjustment for subsequent human trials [142]. Celastrol, a candidate agent for improving cardiac hypertrophy, is limited in in vivo application by poor biocompatibility and low bioavailability, while ROS-responsive nanomicelles encapsulating celastrol show excellent stability, a high stimulus-responsive release rate and superior biocompatibility, and effectively boost the therapeutic activity of celastrol against cardiac hypertrophy [105]; this nanomicelle system has undergone comprehensive in vivo pharmacokinetic and biocompatibility evaluations in mice, with its targeted regulatory effect on the NF-κB pathway verified experimentally, which supports the clinical translation of celastrol, whereas the lack of large animal model data and unclear human metabolic kinetics currently restrict its rapid clinical advancement [105]. PPS-PEG-RA@TA micelles possess prominent anti-inflammatory potential against atherosclerosis and excellent biocompatibility without severe toxic side effects [94]; intravenous administration of this system in mice has achieved significant aortic plaque reduction and confirmed long-term in vivo biosafety, granting it preliminary preclinical translational value, while its targeting ability to human atherosclerotic lesions and the adaptability of its synergistic drug ratio for different patient populations still demand further in-depth research [94]. Exosomes derived from baicalin-pretreated mesenchymal stem cells can inhibit the progression of atherosclerosis by regulating the SIRT1/NF-κB signaling pathway [25]; these modified exosomes exert anti-AS effects in mouse models of high-fat diet-induced atherosclerosis, and their endogenous origin confers unique biocompatibility advantages for clinical translation, yet low exosome yield, complex separation and purification procedures, and insignificant therapeutic superiority over unmodified mesenchymal stem cell-derived exosomes in animal models have become key factors restricting their clinical transformation [25].

Collectively, these advanced delivery platforms have made substantial progress in solving the core challenges that hinder the clinical translation of natural NF-κB inhibitors and related bioactive agents for cardiovascular disease therapy.

### 6.3. Combination of Therapeutic Strategies

Combination approaches using more than one compound are also studied to treat CVDs. Artemisinin combined with allicin inactivates the NF-κB signaling cascade, reduces cardiac dysfunction and myocardium fibrosis [13]. A combination of tanshinone IIA and hydroxy safflower yellow A reduces cerebral infarct volume and neurological deficits, improves cerebral histopathological damage and inhibits the expressions of TNF-α and IL-6 [93]. In contrast to single-agent therapy, the combination of syringin with tilianin improves cardiac function and DCM markers, decreases the expression of NLRP3/IL-6/IL-1β/TNF-α, and reduces oxidative stress. It decreases caspase-3 and Bax/Bcl2 expression. The combination method makes crosstalk between TLR4/NF-κB/NLRP3 and PGC1α/SIRT3/mitochondrial pathways to improve the diabetes-induced cardiac functional, biochemical and histopathological injury [128].

## 7. Challenges and Future Directions

### 7.1. Current Research Limitations

Current studies of natural products targeting the NF-κB signaling pathway to treat CVDs retain key problems. The mechanisms of CVDs are complex, but, fortunately, natural products with multi-targets contribute to solving the problem. These compounds participate in crosstalk such as between Nrf2/HO-1, SIRT1, MAPK, and PI3K/Akt. However, it also makes it difficult to confirm the precise treatment target. To date, there is still a lack of relevant in-depth studies, including on effect mechanisms or drug combinations.

Additionally, it is difficult to fully replicate clinical disease phenomena via in vitro or animal models with different genetic backgrounds and pathophysiological characteristics. This limits the predictive validity of the actual therapeutic efficacy. Clinical trials have strict requirements and high costs, coupled with their insufficient scale, which limits drug research and development.

### 7.2. Future Research Directions

To overcome these difficulties, lots of work remains to be done. First, further studies should be investigated. For example, the research should be focused on the pathogenesis of cardiovascular diseases, the mechanisms of natural products treating cardiovascular diseases and more forms of drug combinations. To precisely identify the target of treating CVDs, single-cell transcriptomics, spatial omics, network pharmacology or other technologies should be applied.

Second, computer-aided drug design and medicinal chemistry should be considered to optimize the structure of compounds, aiming to improve their target selectivity, metabolic stability and oral bioavailability.

Third, more preclinical models should be established to better simulate clinical conditions as much as possible. Large-scale clinical trials with reasonable cost control should be carried out to confirm the effects of natural products on treating CVDs using NF-κB pathway biomarkers.

## 8. Conclusions

The NF-κB signaling pathway represents a central nexus in cardiovascular disease pathogenesis, orchestrating inflammation, oxidative stress, and tissue remodeling across diverse cardiac and vascular pathologies. Natural products—including flavonoids, terpenoids, alkaloids, and polysaccharides—offer a structurally diverse pharmacopeia of NF-κB modulators that exert cardioprotective effects through multifaceted mechanisms, such as inhibiting IKK activation, IκB degradation, p65 nuclear translocation, and crosstalk with complementary antioxidant pathways (e.g., Nrf2/HO-1 and SIRT1).

Mechanistically, natural products have shown plausible and reproducible effects on the NF-κB pathway in preclinical models, with consistent data supporting their efficacy against major cardiovascular conditions like atherosclerosis, myocardial ischemia/reperfusion injury, and hypertensive remodeling. These studies have mapped out the multifaceted molecular mechanisms through which natural products target NF-κB signaling, confirming their potential as cardioprotective agents—at least in the lab.

Despite promising preclinical results, significant gaps remain. For one, dose equivalence across different formulations and species has not been fully worked out. Long-term safety profiles, including possible off-target effects, are still murky. We also lack systematic data on how these natural product-based NF-κB inhibitors might interact with standard cardiovascular drugs, and their long-term clinical outcomes in actual patients remain anyone’s guess.

To move these compounds from bench to bedside, several priorities stand out. First, we need standardized endpoints for both preclinical and clinical studies so results can be compared across labs and trials. Second, the bioavailability issues and complex multi-target pharmacokinetics that plague natural products demand thorough PK/PD investigation. Third, well-designed, large-scale RCTs are essential to establish real efficacy and safety in cardiovascular patients. Beyond that, refining structure–activity relationships, developing smarter nano-delivery systems for targeted administration, and integrating biomarker-guided validation will be crucial for breaking through current translational bottlenecks.

If we can combine mechanistic rigor with innovative pharmaceutical approaches while filling these knowledge gaps, natural product-based NF-κB inhibitors could find their place as complementary therapies in cardiovascular care. But their full clinical value still needs to be proven through hard clinical data.

## Figures and Tables

**Table 1:** The effect of natural products on NF-κB signaling pathway in cardiovascular diseases.

Types of Compounds	Compound Name	Derived from Plants	Types of Diseases	Mechanism	Ref.
Flavonoids	Isoliquiritigenin	Genus Glycyrrhiza	Subarachnoid hemorrhage	Inhibits the expression of TNF-α, IL-6, IL-1β, IL-18, NLRP3, ASC, and caspase-1; inhibits NF-κB p65 expression and its transport	[36]
	Luteolin	Not mentioned	Hypertension; preeclampsia	Inhibits PI3K/Akt/NF-κB, reduces inflammation and ROS production	[7,18]
	Apigenin	Not mentioned	Atherosclerosis; inflammatory diseases	Inhibits NLRP3 inflammasome activation and reduces ROS; inhibits NF-κB p65 nuclear translocation	[8]
	Kaempferol	Not mentioned	Atherosclerosis; hypertension; cardiovascular; dysfunction	Inhibits Piezo1 channels; suppresses ROS production; blocks NF-κB; activates Nrf2/HO-1	[10,24,51]
	Quercetin	Not mentioned	Cardiotoxicity; intimal hyperplasia; CI/RI; PAH; vascular inflammation	Reduces oxidative stress; inhibits inflammatory cytokine production and NF-κB signaling	[21,26,31,33,52,53]
	Baicalin	*Scutellaria baicalensis* Georgi	Atherosclerosis; cardiotoxicity; deep vein thrombosis; cardiac fibrosis	Regulates SIRT1/NF-κB; inhibits TLR4/NF-κB; modulates GRK2/AT1R/MasR; activates Wnt/β-catenin	[25,34,54,55]
	Norwogonin	Not mentioned	Acute lung injury (related vascular diseases)	Inhibits Src/AKT1/NF-κB pathway; reduces inflammation and lung injury	[17]
	Amentoflavone	Not mentioned	Neuroinflammation	Inhibits TLR4/MyD88/NF-κB, activates Nrf2/HO-1 pathway	[29]
	Galangin	Not mentioned	Cyclophosphamide-induced cardiotoxicity	Suppresses NF-κB; activates SIRT1/Nrf2/PGC-1α/TFAM	[56]
	Morin hydrate	Not mentioned	Atherosclerosis; endothelial inflammatory responses	Inhibits PI3K/Akt1/NF-κB; suppresses inflammation	[57]
	Fisetin	Not mentioned	Obesity cardiomyopathy	Inhibits NF-κB/MAPK; reduces cardiac inflammation, hypertrophy and fibrosis	[38]
	Alpinetin	Cardamom seeds	Acute myocardial infarction	Inhibits TLR4/MyD88/NF-κB; relieves cardiac inflammation and remodeling	[58]
	(2S)-5-methoxy-6-methyl-flavan-7-ol	*Sanguis draconis*	Atherosclerosis	Inhibits monocyte-endothelial adhesion; downregulates ICAM-1; regulates NF-κB/IκBα and JNK/Stat3 pathways	[59]
	Dihydromyricetin	Vine tea	High glucose-induced endothelial dysfunction	Reduces oxidative stress; regulates HIF-1α/ROR2/NF-κB pathway	[60]
	4-Hydroxychalcone	Not mentioned	Atrial fibrillation	Inhibits IKK-NF-κB signaling pathway; reduces inflammation, OS, and fibrosis	[61]
	Pelargonidin	Not mentioned	Endothelial inflammation	Inhibits NF-κB; downregulates COX-2; inhibits inflammation	[62]
	Epigallocatechin-3-gallate	Not mentioned	Ethanol-induced endothelial injury	Inhibits NF-κB translocation; activates Nrf2 pathway	[63]
	(-) Epicatechin	Not mentioned	Myocardial infarction	Inhibits NF-κB inflammatory signaling pathway	[64]
	Procyanidin B2	Not mentioned	Hyperglycemia-induced retinal microvascular dysfunction	Regulates redoxosomes/NF-κB; reduces oxidative stress and inflammation	[65]
	Scutellarin	Not mentioned	Ischemic stroke brain injury	Inhibits p65 and p38 and inflammation; reduces oxidative stress	[32]
	Wogonoside	*Scutellaria baicalensis*	Atherosclerosis	Inhibits TLR4/NF-κB; reduces inflammation and oxidative stress	[35]
	Breviscapine	*Erigeron breviscapus* (Vant.) Hand.-Mazz.	Cerebral ischemia-reperfusion injury	Inhibits TLR4/MyD88/NF-κB pathway	[66]
	Liquiritin	Licorice	Cardiac fibrosis; heart failure	Antioxidant; reduces inflammation; regulates AMPK/SIRT1/NF-κB pathway	[67,68]
	Hydroxysafflor yellow A	*Carthamus tinctorius* L.	Cerebral ischemia-reperfusion injury	Reduces inflammation; improves the interactions between SIRT1 and HMGB1; regulates NF-κB pathway	[20]
	Icariside II	*Herba epimedii*	Ischemic stroke	Activates Nrf2; reduces oxidative stress, inflammation, and ferroptosis; regulates OXPHOS/NF-κB pathway	[69]
	Kaempferol-3-O-rutinoside	Lu’an GuaPian tea	Acute myocardial infarction-induced ventricular remodeling	Inhibits NF-κB/NLRP3/Caspase-1 pathway	[70]
	Linarin	Not mentioned	Cardiotoxicity	Suppresses TLR4/HMGB1/RAGE/NF-κB axis; lowers pro-inflammatory cytokines and inflammation	[71]
	Diosmin	Not mentioned	Cardiotoxicity	Antioxidant; blocks NF-κB activation; reduces inflammatory cytokines	[72]
	Hesperidin	Citrus fruits	Kawasaki disease; pulmonary arterial hypertension	Blocks NF-κB activation; lowers pro-inflammatory cytokines; reduces vascular remodeling; protects endothelial cells from apoptosis.	[12,73]
	Naringin	Not mentioned	Radiation-induced heart disease	Activates Sirt1; blocks NF-κB; reduces endoplasmic reticulum stress; lowers fibrosis	[74]
	Phloridzin	Not mentioned	Diabetic cardiomyopathy	Inhibits MyD88/NF-κB; activates Nrf2/GPX4; reduces inflammation; counters oxidative stress; prevents ferroptosis	[75]
	Astilbin	Not mentioned	Sepsis-induced cardiac injury	Activates NRF2/HO-1; inhibits TLR4/NF-κB; reduces inflammation and oxidative stress	[76]
	Genistein-3′-sodium sulfonate	Not mentioned	Ischemic stroke	Inhibits TLR4/NF-κB; regulates astrocyte polarization; improves brain functional rehabilitation	[77]
	*Lycium ruthenicum* Murray Anthocyanins	*Lycium ruthenicum* Murray	Aging-related disorders including cardiovascular diseases	Decreases ROS level; activates SIRT1; inhibits p53, p21	[78]
Terpenoids	Sweroside	Not mentioned	Atherosclerosis	Binds MAP4K4; blocks NF-κB; reduces inflammation; alleviates endothelial injury	[39]
	Catalpol	*Rehmannia*	Atherosclerosis	Reduces ROS; inhibits NF-κB; prevents EndMT	[79]
	Aucubin	Not mentioned	Myocardial ischemia-reperfusion injury	Activates STAT3; inhibits NF-κB nuclear translocation; blocks HMGB1 release; reduces inflammation and apoptosis	[11]
	Rehmannioside A	*Rehmannia*	Hypertensive nephropathy	Inhibits AT1R/MAPK14/IL-17; reduces renal inflammation and fibrosis	[80]
	Santonin	Not mentioned	Doxorubicin-induced cardiotoxicity	Inhibits TLR4/NF-κB; activates Nrf2/HO-1; reduces caspase-3; lowers inflammation and oxidative stress	[81]
	Atractylenolide III	*Atractylodes macrocephala*	Cerebral ischemia-reperfusion injury	Activates PI3K/Akt; inhibits NF-κB; reduces neuroinflammation; preserves blood–brain barrier integrity	[82]
	Isolinderalactone	*Lindera aggregata*	Atherosclerosis	Inhibits NF-κB; reduces macrophage inflammation; lowers oxLDL uptake	[40]
	Lactucopicrin	*Cichorium intybus* L.	Chronic inflammatory diseases; atherosclerosis	Inhibits NF-κB; reduces inflammatory cytokines; blocks p65 transport	[83,84]
	Vernodalin	Not mentioned	Myocardial infarction	Inhibits NF-κB; activates AMPK and eNOS; enhances VEGF-B; reduces inflammation and injury	[85]
	Costunolide	Costus root	Atherosclerosis	Binds Cys179 on IKKβ; inhibits NF-κB/p65 activation; reduces inflammation	[16]
	Valencene	Not mentioned	Myocardial infarction	Inhibits NF-κB; reduces cardiac hypertrophy; lowers oxidative stress; limits infarct size	[86]
	Nerolidol	Aromatic plants	Doxorubicin-induced chronic cardiotoxicity	Activates PI3K/Akt; enhances Nrf2/HO-1; inhibits NF-κB/MAPK; reduces inflammation and pyroptosis	[87]
	Artesunate	*Artemisia annua*	Cardiac hypertrophy; cerebral ischemia; cardiac hypertrophy	Inhibits NF-κB; activates SIRT1; reduces inflammation; alleviates cardiac and cerebral injury	[13,28,88,89]
	Cedrol	Ginger	Acute ischemic stroke	Binds ERβ; inhibits NF-κB; suppresses microglial neuroinflammation; reduces brain infarction	[90]
	Tanshinone IIA	*Salvia miltiorrhiza*	Atherosclerosis; cerebral ischemia-reperfusion injury	Downregulates MAPKs/NF-κB/TLR4; reduces inflammation and lipid accumulation	[91,92,93,94]
	Oridonin	*Rabdosia rubescens*	Cardiac allograft rejection	Inhibits NF-κB/NLRP3; reduces IL-1β and IL-18; expands regulatory T cells	[95]
	Neoandrographolide	Not mentioned	Myocardial ischemia-reperfusion injury	Inhibits NF-κB; reduces Bax/caspase-3, inflammation and apoptosis; increases Bcl-2	[96]
	Cornel iridoid glycosides	*Cornus officinalis* Sieb. et Zucc.	Cerebral ischemia-reperfusion injury	Inhibits TLR4/MyD88; blocks NF-κB nuclear translocation; reduces microglia aggregation; lowers neuroinflammation	[97]
	Paeoniflorin	Not mentioned	Pulmonary arterial hypertension	Inhibits TAK1; blocks MAPK/NF-κB; prevents EndMT; reduces vascular remodeling and inflammation	[98]
	Ovatodiolide	*Anisomeles indica*	Cerebral ischemia-reperfusion injury	Activates SIRT1; inhibits NF-κB; reduces microglial inflammation; prevents neuronal apoptosis	[99]
	Alterbrassicene A	Not mentioned	Calcific aortic valve disease	Binds p65 and inhibits NF-κB phosphorylation; reduces Runx2 and BMP2; prevents valve calcification	[100]
	Carnosic acid	Rosemary	Lipopolysaccharide-induced heart inflammation	Inhibits NF-κB; blocks MAPK activation; enhances CRYAB; reduces inflammatory cytokines	[101]
	Carnosol	Rosemary and sage	Diesel exhaust particles-induced cardiotoxicity	Activates SIRT1; inhibits NF-κB and MAPKs; reduces oxidative stress; prevents DNA damage and apoptosis	[102]
	Lupeol	Not mentioned	Cardiac hypertrophy	Inhibits TLR4; activates PI3K/Akt; blocks NF-κB nuclear translocation; reduces inflammation	[103]
	Madecassic acid	Not mentioned	Cardiomyocyte injury induced by hypoxia reoxygenation	Inhibits inflammatory response and oxidative stress; regulates Nrf2/HO-1/NF-κB signaling pathway	[104]
	Celastrol	*Tripterygium wilfordii*	Cardiac hypertrophy; Renal ischemia-reperfusion injury	Inhibits NF-κB; activates Nrf2/HO-1 and PI3K/AKT; reduces pyroptosis, oxidative stress and inflammation	[105,106]
	Cycloastragenol	*Astragalus* radix	Subarachnoid hemorrhage Ischemic stroke	Activates SIRT1; inhibits acetylation of FoxO1, NF-κB and p53; reduces oxidative stress, neuroinflammation and neuronal apoptosis	[107,108]
	Gypensapogenin I	*Gynostemma pentaphyllum*	Myocardial damage	Inhibits TLR4/NF-κB; blocks NLRP3 activation; reduces fibrosis	[109]
	Fucoxanthin	Not mentioned	Intracerebral hemorrhage	Activates PI3K/Akt; inhibits NF-κB; suppresses M1 polarization; reduces neuroinflammation and neuronal apoptosis	[110]
	Lycopene	Not mentioned	Palmitate-mediated myocardial inflammation; obesity-related heart failure	Inhibits NF-κB; modulates lipid metabolism; enhances antioxidants; reduces inflammatory cytokines	[111]
	β-cryptoxanthin	Not mentioned	Myocardial ischaemia/reperfusion injury	Inhibits NF-κB nuclear translocation; blocks p38 MAPK; reduces TNF-α and IL-6; limits infarct size	[112]
	Astaxanthin	Not mentioned	Isoprenaline-induced myocardial infarction	Inhibits TLR4/NF-κB; reduces TNF-α and oxidative stress	[113]
	Astragaloside IV	*Astragalus membranaceus*	Sepsis-induced cardiac dysfunction; pulmonary ischemia-reperfusion injury; acute myocardial infarction; high-altitude hypoxia-induced cardiac injury	Inhibits IKK/NF-κB; blocks TLR4/MyD88; reduces inflammation; activates CaSR and EGFR-PI3K-AKT pathways	[22,43,114,115]
	Ginsenoside Rb1	*Panax ginseng*	Intracerebral hemorrhage; obesity-related cardiac fibrosis	Inhibits TLR4/NF-κB; regulates GRK2/AT1R/MasR; reduces astrocyte activation and neuroinflammation	[56,116]
	Ginsenoside Rb3	*Panax ginseng*	Cerebral ischemia/reperfusion injury	Inhibits NLRP3/NF-κB; activates GPX4; reduces ferroptosis and neuroinflammation	[117]
	20(R)-ginsenoside Rg3	*Panax ginseng*	Cerebral ischemia/reperfusion injury; stroke	Inhibits TLR4/MyD88; blocks NF-κB p65 phosphorylation; reduces neuroinflammation	[118]
	Ciwujianoside C	*Acanthopanax senticosus*	Cerebral ischemia-reperfusion injury	Activates NNAT; inhibits NF-κB; reduces iron accumulation	[119]
	Ecliptasaponin A	*Eclipta prostrata* (Linn.)	Acute myocardial infarction	Binds HMGB1; inhibits TLR4/NF-κB; reduces IL-6 and TNF-α; decreases infarct size and apoptosis	[120]
	Platycodin D	*Platycodon grandiflorum*	Diabetic retinopathy; Diabetic renal ischemia/reperfusion injury	Inhibits TLR4/MyD88/NF-κB; activates Nrf2/HO-1; enhances AMPK/PINK1/Parkin mitophagy; reduces inflammation and oxidative stress	[121]
	Esculin	*Cortex fraxini*	Septic cardiomyopathy	Binds TLR4; inhibits NF-κB p65 phosphorylation; reduces inflammation, oxidative stress and apoptosis	[48]
	Escin	Not mentioned	Cerebral ischemia-induced intestinal injury	Inhibits p38 MAPK/NF-κB; blocks NLRP3 inflammasome; reduces pyroptosis	[122]
	Asiaticoside	*Centella asiatica*	Atherosclerosis	Binds RhoF and promotes its degradation; inhibits NF-κB/MAPK; reduces macrophage inflammation	[123]
Phenylpropanoids	Plantamajoside	*Plantago asiatica*	Myocardial I/R injury	Activates Akt/Nrf2/HO-1 antioxidant pathway; inhibits NF-κB inflammatory signaling; reduces oxidative stress, inflammation, and apoptosis	[124]
	Rosmarinic acid	Labiatae herbs	Myocardial I/R injury	Suppresses NF-κB pathway and ROS production; reduces inflammation and oxidative stress	[125]
	Forsythoside B	*Forsythia suspensa*	PAH; Kawasaki disease-induced cardiac injury	Activates SIRT1; inhibits NF-κB-p65 and pyroptosis	[126,127]
	Syringin	Not mentioned	Diabetic cardiomyopathy	Inhibits TLR4/NF-κB/NLRP3; regulates PGC1α/SIRT3	[128]
	Isofraxidin	Not mentioned	Cerebral I/R injury	Modulates TLR4/NF-κB; reduces oxidative stress and neuroinflammation	[129]
	Fraxin	Cortex Fraxini	Cerebral I/R injury	Activates PPAR-γ/Nrf2/HO-1; inhibits NF-κB and cell apoptosis	[130]
	Imperatorin	Not mentioned	Ischemic stroke	Inhibits MAPK and NF-κB; reduces neuroinflammation	[41]
	Aesculin	Cortex Fraxini	Myocardial I/R injury	Activates Akt/GSK3β; inhibits NF-κB and NLRP3 inflammasome	[131]
	Urolithin A	Not mentioned	Pulmonary hypertension	Activates AMPK; inhibits NF-κB/NLRP3 and PASMC pyroptosis	[48]
	Phillyrin	*Forsythia suspensa*	Norepinephrine-induced cardiac hypertrophy	Suppresses p38/ERK1/2 MAPK and AKT/NF-κB pathways; reduces inflammatory response, ROS production, and cardiomyocyte hypertrophy	[46,132]
	Magnolin; Aschantin; Fargesin	*Magnoliae flos*	Inflammatory diseases; cardiovascular diseases; cerebral I/R injury	Inhibits NF-κB, TLR4; reduces neuroinflammation and oxidative stress	[133,134]
	Syringaresinol	Not mentioned	Myocardial infarction	Multiple targets modulations; reduces inflammation, fibrosis, and apoptosis	[135]
	Podophyllotoxin (exceptional case: induce cardiotoxicity)	Not mentioned	Cardiotoxicity	Inhibits SIRT1/AMPK/PGC-1α/PPAR; increases p-IKK and p-IκBα; leads to the nuclear translocation of NF-κB p65 from the cytosol; induces inflammation	[136]
	Schisantherin A	*Schisandra chinensis* fruits	Acute myocardial infarction	Activates PI3K-AKT/Nrf2; inhibits TLR4/MAPK/NF-κB; reduces oxidative stress and inflammation	[137]
	Pinoresinol diglucoside	*Eucommia ulmoides*, *Styrax* sp., *Forsythia suspensa*	Cardiac hypertrophy	Inhibits Akt/mTOR/NF-κB; reduces inflammation and fibrosis	[138]
Alkaloids	Berberine	Not mentioned	Septic cardiomyopathy; calcified aortic valve disease	Inhibits TLR4/Smad1/5/8 and NF-κB	[19,139]
	Tetrandrine	Not mentioned	Hypertensive heart failure; Atherosclerosis	Inhibits MAPK/NF-κB and Wnt5a/Ror2/ABCA1/NF-κB pathways	[4,140]
	Colchicine	*Colchicum autumnale*	Atherosclerosis; myocardial infarction	Inhibits NLRP3; suppresses CCR7/NF-κB	[23,43,141,142]
	Matrine	*Sophora flavescens*	Pulmonary hypertension; heart transplantation acute rejection	Inhibits ROS/ERK/NF-κB pathway	[143,144]
	Gramine	Wide variety of raw plants	Sepsis-induced myocardial dysfunction	Binds NF-κB p105; blocks its ubiquitination and processing to p50 subunit	[30]
	Lycorine	*Lycoris radiata*	Cardiac remodeling	Blocks PI3K-AKT/NF-κB pathway; reduces inflammation and remodeling	[145]
	Piperlongumine	*Piper longum* L.	Viral myocarditis	Inhibits NF-κB; suppresses pyroptosis and inflammation	[146]
	Tetrahydropalmatine	*Corydalis yanhusuo*	Limb ischemia-reperfusion-induced acute lung injury	Inhibits TLR4/NF-κB/NLRP3; promotes M1-to-M2 macrophage polarization	[147]
	Boldine	*Peumus boldus*	Cardiac fibroblast inflammation	Inhibits SGK1 and NF-κB; reduces inflammatory responses	[148]
	Anatabine	Not mentioned	Hypertension	Blocks NF-κB/NLRP3/caspase-1 pyroptosis pathway; reduces sympathetic drive	[149]
	Anisodamine (654-1/654-2)	Not mentioned	Septic shock-induced myocardial dysfunction	Activates PI3K-AKT; inhibits NF-κB/NLRP-3	[150]
	Rutaecarpine	*Evodia rutaecarpa*; *Tetradium ruticarpum*	Inflammatory diseases; pulmonary thrombosis	Inhibits NF-κB and ERK/p38 MAPK pathways	[151,152]
	Oxymatrine	*Sophora flavescens*	Isoproterenol-induced heart failure	Blocks TLR4/NF-κB and MAPK pathways; reduces inflammation and cardiac injury	[153]
	Vincristine	Not mentioned	Isoprenaline-induced cardiac hypertrophy	Inhibits ROS/NO/NF-κB signaling; reduces oxidative and inflammatory stress	[47]
	Corynoline	Not mentioned	Ang II-induced hypertensive heart failure	Promotes PPARα-p65 binding; inhibits NF-κB	[154]
	Koenigicine	Not mentioned	Isoproterenol-induced myocardial infarction	Modulates NF-κB/HO-1/NQO-1; enhances antioxidant; reduces inflammation	[155]
	Leonurine	*Leonurus japonicus* Houtt.	Ang II-induced hypertensive cardiac injury	Inhibits MAPK and NF-κB activation; prevents hypertrophy and fibrosis	[156]
	Capsaicin	chili pepper	Hypertension; cardiac hypertrophy	Activates SIRT1; suppresses NF-κB/MAPKs/AKT/ERK1/2	[44,50]
Quinones	Thymoquinone	Not mentioned	Vasoconstriction injury; cardiotoxicity	Inhibits NF-κB; reduces ROS/RNS; downregulates AQP4 and inflammatory proteins	[157,158]
	Shikonin	Not mentioned	Cerebral I/R injury	Inhibits NOD2/RIP2/NF-κB; modulates microglia polarization; reduces neuroinflammation	[159]
	Dihydrotanshinone I	Not mentioned	Calcific aortic valve disease	Inhibits SMAD1/5/8/NF-κB/ERK; reduces valve interstitial cell calcification	[160]
	Emodin	Not mentioned	Atherosclerosis	Suppresses TLR4/MyD88/NF-κB; inhibits NLRP3/GSDMD; reduces inflammation	[161]
	Aloe-emodin	Sanhua Decoction; Aloe and rhubarb	Subarachnoid hemorrhage; ischemic stroke	Inhibits NF-κB; upregulates PI3K/AKT/mTOR; reduces inflammation	[162,163]
	Physcion	Not mentioned	Cerebral I/R injury	Inhibits TLR4/NF-κB; reduces oxidative stress and neuronal apoptosis	[164]
Steroids	Dioscin	Not mentioned	Sepsis-induced cardiomyopathy	Reduces cardiac inflammation and oxidative stress; blocks TLR4/MyD88/p65 signaling	[165]
	Cycloastragenol	*Astragalus membranaceus*	Endothelial inflammation and acute lung injury	Inhibits inflammation; inactivates NF-κB pathway	[166]
	Saponins from Allium macrostemon Bulbs	*Allium macrostemon*	Atherosclerosis	Inhibits CD36, ox-LDL, and lipid endocytosis; regulates NF-κB/NLRP3	[167]
	Steroid from Solidago canadensis	*Solidago canadensis*	LPS-induced inflammation (major risk factor for cardiovascular disease including atherosclerosis)	Activates AMPK; inhibits NLRP3 inflammasome and NF-κB	[168]
Polyphenols	Resveratrol	Grapes, red wine	Hypertension; vascular dysfunction; obesity-induced vasculopathy; myocardial infarction	Inhibits TLR4/MyD88/TNF-α/HIF-1α/iNOS/NF-κB; activates AMPK/SIRT1; lowers blood lipid	[169,170,171,172]
	Curcumin	*Curcuma longa*	Diabetic cardiomyopathy inflammation and remodeling; cardiotoxicity;	Reduces inflammation; decreases Apelin expression; downregulates Rac1/TWEAK/Fn14/NF-κB	[49,173,174,175]
	Salidroside	*Rhodiola*	Pulmonary hypertension	Suppresses AhR/NF-κB; activates Nrf2/HO-1; inhibits PAEC apoptosis	[176]
	Velutin	Not mentioned	Abamectin-induced cardiotoxicity	Regulates JAK1/STAT3, NF-κB, Nrf-2/Keap-1 pathways; reduces oxidative stress and apoptosis	[177]
	Oleuropein	Olive plant	Pancreatic ischemia reperfusion injury	Inhibits HMGB1/NF-κB; suppresses oxidative stress and inflammatory cytokines	[178]
	Total xanthones from *Gentianella acuta*	*Gentianella acuta*	Acute myocardial infarction	Inhibits BRD4/TLR4/NF-κB/NLRP3; reduces cardiomyocyte pyroptosis	[179]
	Salvianolic acid A	*Salvia miltiorrhiza*	Doxorubicin-induced cardiotoxicity	Inhibits NFKB1; downregulates lncRNA PVT1; blocks apoptosis	[180]
	Gastrodin	*Gastrodiae elata* Blume	Atherosclerosis and vascular inflammation	Reduces inflammation; inhibits TLR4/NF-κB	[181]
	Polydatin	Not mentioned	Diabetic cardiomyopathy	Inhibits caveolin 1-dependent NF-κB; reduces inflammatory fibrosis	[182]
Saccharides	L-fucose	Not mentioned	Obesity-related cardiac injury	Inhibits TLR4/MyD88/NF-κB; reduces inflammation, pyroptosis and mitochondrial injury	[183]
	Astragalus polysaccharides	*Astragalus membranaceus*	Doxorubicin cardiomyopathy; transport stress-induced cardiac injury	Activates AMPK and PPAR-γ; inhibits NLRP3, cGAS-STING, and NF-κB; reduces oxidative stress, inflammation, and fibrosis	[184,185]
	*Poria cocos* polysaccharides	*Poria cocos*	Atherosclerosis	Inhibits TLR4/NF-κB activation; lowers inflammatory cytokines and lipids; reduces oxidative stress	[186]
	*Polygonatum cyrtonema* Hua polysaccharide PCP1	*Polygonatum cyrtonema* Hua	Atherosclerosis	Inhibits CD36/MSR1 and TLR4/NF-κB	[187]
	Water-soluble polysaccharides from *citri reticulatae* pericarpium	*Citrus reticulata* (dried peel)	LCWE-induced endothelial dysfunction; coronary arteritis	Targets TLR2; inhibits NF-κB-NLRP3; reduces endothelial barrier injury	[188]
	*Ganoderma lucidum* triterpenoids and polysaccharides	*Ganoderma lucidum*	Atherosclerosis	Inhibits NF-κB/LOX-1 and Notch1/DLL4; reduces oxidative stress and inflammation	[189]
Tannins	Punicalagin	*Punica granatum* L.	Isoproterenol-induced myocardial infarction; LPS-stimulated inflammation	Inhibits p38 MAPK and NF-κB phosphorylation; decreases iNOS, COX-2, IL-6 and TNF-α; upregulates Nrf2/HO-1	[190,191]
	Corilagin	*Phyllanthus urinaria* L.	Atherosclerosis	Inhibiting the LOX-1/MyD88/NF-κB pathway	[192]
Volatile oil	Carvacrol	Aromatic plants, fragrance essential oils	LPS-induced myocardial dysfunction	Inhibit TLR4/MyD88/NF-κB and NLRP3 inflammasome; reduces inflammation	[193]
Fatty Acids	Lauric acid	Coconut oil	Doxorubicin-induced cardiotoxicity	Inhibits NF-κB p65; reduces oxidative stress and inflammation	[194]
Retinoids	All-trans-retinoic acid	Not mentioned	Middle cerebral artery occlusion-induced cerebral injury	Inhibits TLR4/NF-κB; reduces inflammation	[195]
Traditional Chinese Medicine Combination	Baicalin-Geniposide combination	Not mentioned	Chronic cerebral ischemia and concomitant kidney injury	Increases HIF-1α/EPO; reduces NF-κB phosphorylation and pro-inflammatory factors	[196]
Traditional Chinese Medicine Combination	Beta-caryophyllene-L-arginine combination	Not mentioned	Diabetic cardiomyopathy	Reduces cardiac inflammation and oxidative stress; inhibits NF-κB	[197]
Traditional Chinese Medicine Decoction	Guyuan Jiannao Decoction (GYND)	*Rehmannia glutinosa*, *Cornus officinalis*, *Cistanche deserticola*, etc.	Cerebral small vessel disease	Upregulates PI3K/AKT; inhibits NF-κB	[198]
Traditional Chinese Medicine Decoction	Taohong Siwu Decoction	Baishao (*Radix Paeoniae Alba*), Shudihuang (*Radix Rehmanniae Praeparata*), Danggui (*Radix Angelicae Sinensis*), Chuanxiong (*Rhizoma Chuanxiong*), Taoren (*Semen Persicae*) and Honghua (*Flos Carthami*).	Atherosclerosis	Inhibits TLR4/MyD88/NF-κB; reduces inflammation	[199]
Traditional Chinese Medicine Pills	Gegen Qinlian Pills	*Pueraria montana* var. *lobata* (Willd.) *Sanjappa & Pradeep* (Gegen), *Scutellaria baicalensis* Georgi (Huangqin), *Coptis chinensis* Franch. (Huanglian) and *Glycyrrhiza uralensis* Fisch. (Gancao)	Carrageenan-induced thrombosis	Inhibits HMGB1/NF-κB/NLRP3; reduces inflammation	[200]
Traditional Chinese Medicine Injection	Xueshuantong Injection	*Panax notoginseng*	Cerebral microcirculation disorder (middle cerebral artery occlusion/reperfusion)	Inhibits JNK/JAK2/STAT3 and NF-κB; reduces cerebral inflammation; protects microvascular structure and function	[201]

This comprehensive table systematically categorizes natural products into nine chemical classes (flavonoids, terpenoids, phenylpropanoids, alkaloids, quinones, steroids, polyphenols, saccharides, tannins, other compounds, and compound traditional Chinese medicine formulas and prescriptions) and summarizes their therapeutic applications in various cardiovascular disease (CVD) models. For each compound, the table specifies the botanical origin, specific disease indication, precise molecular target within the NF-κB pathway, and key literature reference.

## Data Availability

No new data were created or analyzed in this study. Data sharing is not applicable.

## Abbreviations

The following abbreviations are used in this manuscript:

Abbreviation	Full Name
4HCH	4-Hydroxychalcone
AF	Atrial fibrillation
AGEs	Advanced glycation end products
Akt	Protein kinase B
AMI	Acute myocardial infarction
Ang II	Angiotensin II
ANP	Atrial natriuretic peptide
APS	Astragalus polysaccharides
ART	Artesunate
ATL III	Atractylenolide III
Ba-exos	Baicalin-pretreated mesenchymal stem cell-derived exosomes
BBB	Blood–brain barrier
BC/GD	Baicalin and geniposide combination
BNP	Brain natriuretic peptide
CAG	Cycloastragenol
CaSR	Calcium-sensing receptor
CAT	Catalase
CAVD	Calcific aortic valve disease
CI/RI	Cerebral ischemia-reperfusion injury
Col@PSVs	Colchicine phosphatidylserine-exposing nanovesicles
CP-0	Citri Reticulatae Pericarpium polysaccharide CP-0
CSVD	Cerebral small vessel disease
CTD	Costunolide
CVDs	Cardiovascular Diseases
DCM	Diabetic cardiomyopathy
DCNPs	Diosmin-loaded chitosan nanoparticles
DOX	Doxorubicin
EGFR	Epidermal growth factor receptor
ERK	Extracellular signal-regulated kinase
ET-1	Endothelin-1
GAD67	67-kDa isoform of glutamate decarboxylase
GQP	Gegen Qinlian Pills
GR	Glutathione reductase
GSH-px	Glutathione peroxidase
GSS	Genistein-3′-sodium sulfonate
GYJND	Guyuan Jiannao Decoction
Gyp I	Gypensapogenin I
HDL-C	High-density lipoprotein cholesterol
HMGB1	High mobility group box 1
HO-1	Heme oxygenase-1
HSYA	Hydroxysafflor yellow A
HUVECs	Human umbilical vein endothelial cells
I/R	Ischemia/reperfusion
I/R injury	Ischemia/reperfusion injury
IDE	Insulin-degrading enzyme
IKK	IκB kinase
IL-1β	Interleukin-1β
IL-6	Interleukin-6
ISL	Isoliquiritigenin
IκB	Inhibitor of κB
JNK	c-Jun N-terminal kinase
KPF@MM-NPs	Kaempferol biomimetic nanoparticles
LA	Lauric acid
LDH	Lactate dehydrogenase
LDL-C	Low-density lipoprotein cholesterol
LIN	Linarin
LPS	Lipopolysaccharide
MAPK	Mitogen-activated protein kinase
MCAO	Middle cerebral artery occlusion
MDA	Malondialdehyde
MF	Myocardial fibrosis
MI	Myocardial infarction
MIRI	Myocardial ischemia/reperfusion injury
MMP	Matrix metalloproteinase
MyD88	Myeloid differentiation primary response 88
NF-κB	Nuclear factor κB
NIK	NF-κB-inducing kinase
NLRP3	NOD-like receptor family pyrin domain containing 3
NO	Nitric oxide
Nrf2	Nuclear factor erythroid 2-related factor 2
NVU	Neurovascular unit
NWG	Norwogonin
OLP	Oleuropein
ox-LDL	Oxidized low-density lipoprotein
p38	p38 MAPK
PAH	Pulmonary arterial hypertension
PCP	*Poria cocos* polysaccharides
PDG	Pinoresinol diglucoside
PE	Preeclampsia
PI3K	Phosphatidylinositol 3-kinase
PPS-PEG-RA@TA	Rosmarinic acid-functionalized micelles
PVN	Paraventricular nucleus
Qu-PEG NS	Quercetin-polyethylene glycol nanosuspension
RAGE	Receptor for advanced glycation end products
RIHD	Radiation-induced heart disease
RIRI	Renal ischemia-reperfusion injury
ROS	Reactive oxygen species
SAH	Subarachnoid hemorrhage
SAMB	Saponins from *Allium macrostemon* bulbs
SHRs	Spontaneously hypertensive rats
SIC	Sepsis-induced cardiomyopathy
SIRT1	Sirtuin 1
SOD	Superoxide dismutase
STAT3	Signal transducer and activator of transcription 3
STS	Sodium tanshinone IIA sulfonate
SYR	Syringaresinol
TAC	Transverse aortic constriction
TC	Total cholesterol
TG	Triglyceride
TGF-β1	Transforming growth factor beta 1
THP	Tetrahydropalmatine
TLR4	Toll-like receptor 4
tMCAO	Transient middle cerebral artery occlusion
TNFR1	Tumor necrosis factor receptor 1
TNFR2	Tumor necrosis factor receptor 2
TNF-α	Tumor necrosis factor-α
VCAM-1	Vascular cell adhesion molecule 1
VCR	Vincristine
VHPK-PLGA@COL	VHPK peptide-modified PLGA nanoparticles encapsulating colchicine
VMC	Viral myocarditis
VR	Ventricular remodeling
XST	Xueshuantong
